# Adaptive control strategies for button motor actuated insect scale flapping wing MAV mechanisms

**DOI:** 10.1038/s41598-025-13834-2

**Published:** 2025-08-05

**Authors:** Spoorthi Singh, Meet Hitesh Jain, Kanishk Kaushal, Mohammad Zuber, Ernnie Illyani Basri, Kamarul Arifin Ahmad, Sharul Sham Dol, Vishnu G. Nair

**Affiliations:** 1 Department of Mechatronics, Manipal Institute of Technology, Manipal Academy of Higher Education, Manipal, Karnataka 576104 India; 2 Department of Aerospace Engineering, Faculty of Engineering, Universiti Putra Malaysia, UPM Serdang, 43400 Selangor, Selangor Malaysia; 3 Department of Aeronautical and Automobile Engineering, Manipal Institute of Technology, Manipal Academy of Higher Education, Manipal, Karnataka 576104 India; 4 Department of Electronics and Communication Engineering, Manipal Institute of Technology, Manipal Academy of Higher Education, Manipal, Karnataka 576104 India; 5 Aerospace Malaysia Research Centre (AMRC), Universiti Putra Malaysia, UPM Serdang, 43400 Selangor, Selangor Malaysia; 6 Putra Tech Park, Universiti Putra Malaysia, UPM Serdang, 43400 Selangor, Selangor Malaysia; 7 Department of Mechanical and Industrial Engineering, Abu Dhabi University, 59911 Abu Dhabi, UAE

**Keywords:** Aerospace engineering, Mechanical engineering

## Abstract

The development of Flapping Wing Micro Aerial Vehicles (FWMAVs) has gained significant attention due to their potential for energy-efficient, lightweight, and highly maneuverable flight inspired by nature. This study presents innovative designs and adaptive control strategies for insect-scale FWMAVs, utilizing compact button vibrator motors as actuators for wing flapping. These actuators offer advantages in size, weight, and power efficiency but pose challenges in achieving continuous and controlled motion due to mechanical, control, and durability constraints. The research explores multiple lever alignment configurations using simplified crank-slider mechanisms, driven by single and dual coreless DC motors powered by a 1–3.7 V DC supply. Detailed modeling in SIMSCAPE Multibody and structural movement analysis using Compmech GIM software facilitate the evaluation of variations in flapping frequency, velocity, and acceleration. Advanced control strategies, including Self-Regulatory Fractional Fuzzy Control (SRFFC) and Fractional PID (FPID), are assessed under simulated and real-world conditions to mitigate external disturbances. Additionally, an AI-based disturbance observer is implemented to enhance stability and optimize power efficiency by compensating for environmental disturbances. Performance metrics such as rise time, settling time, overshoot, and integral absolute error (IAE) demonstrate the superior efficiency and disturbance rejection capabilities of SRFFC compared to FPID. Experimental validation and real-time assessments of maneuvering capabilities, including leftward, rightward, and forward movements, further substantiate the proposed strategies. This study underscores the potential of SRFFC-driven designs and modular motor configurations to enhance the performance, control, and applicability of FWMAVs for advanced micro-aerial systems.

## Introduction

When birds or aircraft fly, their wings are positioned at a slight angle, deflecting the air gently downwards, which increases air pressure beneath the wings and decreases it above them. This pressure difference generates lift, a force acting roughly perpendicular to the wing surface, preventing the bird or aircraft from falling. Bird flight can be classified into two types: unactuated flight (gliding and soaring) and actuated flight (flapping and hovering)^1^. Dragonflies and hoverflies utilize asymmetric strokes along an inclined stroke plane. In contrast to normal hovering, asymmetric flapping generates the highest vertical force during the downward stroke. Instead of relying on the full power stroke required for conventional hovering, an inclined stroke plane allows the weight to be supported by aerodynamic drag. Studies indicate that aerodynamic drag contributes approximately 76% of the vertical force generated by asymmetric flapping, highlighting the effectiveness of this mechanism^2^. The modes of motion in any asymmetrical flight can be categorized into longitudinal, lateral, and coupled modes. In 2020, Taylor^3^ emphasized that handling insect-scale flapping-wing vehicles requires an understanding of the trim map, data-driven dynamic mode decomposition, and the entire flight envelope, while also accounting for asymmetric signal sources and structural constraints imposed by actuators. Observations of hovering flapping motion necessitate distinguishing between wings that flap horizontally (symmetric hovering) and those that follow an inclined stroke plane (asymmetric hovering). Hovering fluctuations have been extensively studied due to their frequent occurrence in the insect world. Several kinematic factors, including the angle of attack (AoA), axis of rotational position, and Reynolds number, significantly influence hovering efficiency. However, investigations into asymmetric hovering remain largely confined to biological configurations, with limited research focusing on MAVs that provide descriptive solutions. Nonetheless, integrating asymmetric hovering into flight dynamics is an exciting technique, as lift generation is influenced by both lift and drag, thereby enhancing aerodynamic efficiency^4–8^. Some large birds have wings with extended structures that provide additional lift during the downstroke, as their internal morphology restricts wing rotation. During the upstroke, they employ flexed retrograde motion to minimize drag^1^. This flexing is more pronounced in slow forward flight compared to fast forward flight. This particular form of asymmetric hovering is referred to as the “avian stroke.”

In 2009, Thierry^8^ utilized fourth-order polynomial motion rules to analyze the kinematics of modification in both upstroke and downstroke phases for demonstration. While examining symmetric motion, the leading-edge vortex (LEV) plays a critical role in controlling the vortical flow field during most of the upstroke phase. However, in the asymmetric scenario, the LEV has a finite duration and exerts its influence only when a narrow lift peak occurs. Adjusting the angle of attack from upstroke to downstroke is feasible with this asymmetric configuration, which rotates faster than the symmetric counterpart. The simultaneous rotation and translation actions enhance circulation motion, increasing lift and inducing the Kramer effect. In 2012, Park^9^ demonstrated that asymmetric wing flapping could be effectively achieved without disrupting the in-phase flapping motion. He developed a novel gear mechanism to accomplish this. The aerodynamic efficiencies for generating both vertical lift and horizontal thrust were evaluated using a quasi-steady aerodynamic model and two-dimensional (2D) computational fluid dynamics (CFD) analysis at a Reynolds number of 2500. Using a button vibrator as a motor to actuate flapping in a flapping-wing mechanism is an innovative approach that leverages off-the-shelf components for a unique application. Button vibrators, also known as coin vibration motors, are small, compact devices commonly used in mobile phones for haptic feedback. When integrated into a flapping-wing design, they offer several advantages, including compact size, low power consumption, ease of integration, and affordability. Their small and lightweight nature makes them suitable for applications where space and weight are critical constraints, such as micro air vehicles. Additionally, low power consumption is essential for preserving battery life in portable devices like micro air vehicles. These coin motors are designed for easy integration into various systems due to their standardized form factor and simple electrical connections. Furthermore, button motors are cost-effective, making them beneficial for prototyping and budget-conscious projects. However, certain limitations must be considered. Button vibrators might not be designed for continuous operation as motors, and the mechanical wear and tear associated with continuous flapping motion could impact their lifespan and reliability. Although they consume low power, they might not provide the torque or power output required for more demanding flight scenarios, which could affect the vehicle’s ability to carry heavy loads or operate under challenging conditions. The unique characteristics of button vibrators might necessitate custom mechanical adaptations to effectively convert rotor rotation into the desired wing-flapping motion. In general, flapping aerial vehicle wings generate sufficient lift to support their overall mass in the air. Aerodynamic efficiency can be evaluated in terms of the energy required to maintain altitude or travel a given distance relative to lift output. Positive average aerodynamic lift can be achieved by asymmetrically adjusting the wing pitch angle. However, in 2018, Li^10^ demonstrated that if flapping strokes become asymmetric, the efficiency of lift generation varies significantly, while optimal propulsive efficiency decreases. This results in the wing producing net drag rather than thrust, with downstroke thrust exceeding upstroke thrust in asymmetric pitching.

At present, the Flapping Wing Unmanned Aerial Systems (FWUAS) sector is witnessing the implementation of a sophisti- cated and streamlined fluttering actuation mechanism that utilizes compact and specialized driving technology. By integrating a button vibrator into the design of fluttering wings to function as a motor for initiating flapping, a novel approach is adopted, offering various advantages along with specific considerations. While the proposed design models provide benefits in terms of power, size, and weight, achieving consistent and regulated wing fluttering presents challenges related to mechanics, durability, and control. To address these challenges, thorough prototyping, testing, and exploration of different design alignments using the button vibrator are essential, particularly for achieving the desired flight performance in micro air vehicles and similar applications. The novelty of this research lies in the comprehensive evaluation and comparison of Self-Regulatory Fractional Fuzzy Control (SRFFC) and Fractional PID (FPID) strategies for Flapping Wing Micro Aerial Vehicles (FWMAVs). SRFFC employs adaptive fuzzy logic rules^11,12^, demonstrating superior disturbance rejection and control efficiency compared to FPID^13–15^. The adaptive nature of SRFFC allows for dynamic adjustment of control parameters, improving the system’s ability to handle external disturbances and maintain stability. By dynamically modifying control parameters based on real-time feedback, SRFFC enables precise and efficient control, compensating for external disturbances while minimizing overshoot and settling time. Furthermore, SRFFC optimizes power consumption by reducing control effort, thereby prolonging the operational lifespan of FWMAVs. Simulations and real-time assessments indicate that SRFFC enhances the maneuverability of FWMAVs, facilitating leftward, rightward, and forward movements. The ability to adapt to varying environmental conditions makes SRFFC particularly suitable for applications requiring high reliability and adaptability. Additionally, SRFFC exhibits faster rise times and shorter settling times due to its adaptive control logic, significantly reducing overshoot compared to FPID, thereby improving system stability and overall performance. The lower Integral Absolute Error (IAE) achieved by SRFFC indicates more efficient error correction and superior control. Moreover, the AI-based disturbance observer integrated with SRFFC effectively mitigates the impact of external disturbances, ensuring desired performance^13,16–23^. To address the challenges of dynamic morphology changes and external disturbances in unconventional aerial robots, this study introduces a Radial Basis Function Artificial Neural Network–Based Fast Terminal Sliding Mode Control (RBFANN-FTSMC). The RBFANN element provides adaptive compensation for time-varying dynamics and parameter uncertainties, while the FTSMC component ensures rapid convergence and robustness against disturbances. Together, these elements enable precise trajectory tracking and real-time control of morphing UAVs under varying configurations.^24,25^.

FWMAVs have gained significant attention due to their potential for maneuverability, lightweight design, and energy efficiency, mimicking the flight of insects. However, designing actuation mechanisms that are both efficient and durable remains a challenge, particularly when scaling down insect-size vehicles. This research introduces a novel actuation mechanism that leverages button vibrators as motors to drive the flapping wings in insect-scale FWMAVs. Button vibrators, typically used in mobile devices for haptic feedback, offer a lightweight and compact alternative to traditional motors, reducing overall mass while providing adequate motion for flapping mechanisms. The key innovation of this work lies in utilizing off-the-shelf button vibrators to replace conventional motor-driven systems in flapping wing Unmanned Aerial Systems (FWUAS). This approach not only reduces the size and weight of the FWMAV but also opens avenues for new design architectures. The simplified crank-slider mechanism, driven by a button vibrator, significantly reduces mechanical complexity, allowing for a more compact and efficient system. Additionally, the integration of SRFFC introduces adaptive control strategies, enabling real-time compensation for external disturbances, thereby improving overall stability and control efficiency. A major concern with button vibrators in continuous flapping applications is their longevity. These devices are not traditionally designed for sustained mechanical output, which can lead to mechanical wear and tear over time. To address this, this study includes *extended durability testing* to evaluate the long-term operational capacity of button vibrators under continuous flapping conditions. The tests focus on assessing the impact of mechanical stress and heat on motor lifespan, providing insights into how these actuators can be optimized or adapted for more robust applications in FWMAVs. The study also explores potential design modifications that could enhance the durability of these motors, such as advanced cooling mechanisms or reinforced housing structures. Given the limited power output of button vibrators, efficient power management is crucial for maintaining flight performance over extended periods. This study presents novel *power optimization strategies* that balance energy consumption with performance. Techniques such as regenerative energy recovery during wing downstrokes and adaptive control algorithms for optimizing power distribution are introduced. These strategies are designed to prolong battery life while maintaining adequate lift and thrust, addressing one of the core limitations of small-scale FWMAVs—their limited flight duration due to constrained power resources. In addition to the use of single button vibrators, this research also explores *modular motor configurations*, where multiple button motors drive each wing independently. This modular approach offers enhanced flexibility in maneuverability and control. By allowing for the decoupling of motor inputs to individual wings, the Unmanned Aerial Systems can achieve more precise directional control, particularly in complex environments or during dynamic flight maneuvers. This modular configuration also supports redundancy, which could enhance the reliability of the drone in case of motor failure, making it more resilient in real-world applications^2,4,12,26,27^.

This study conducts a comparative analysis of various lever alignment designs utilized in proposed insect-scale FWMAV propelled by single and double motors during flapping actuation. Miniature, coreless DC motors fueled by a 1–3.7 V DC supply are the selected motors. By utilizing various lever configurations, the proposed actuation mechanisms are connected indirectly to a crank-slider mechanism that is comparatively straightforward. The modeling and control of a slider-crank mechanism design utilizing SIMSCAPE Multibody in MATLAB are described in the paper. Furthermore, simulation validations involve the execution of structural movement analysis in Compmech GIM software^28,29^, which includes both 2D and 3D analysis, in addition to CAD design. Analyzed variations of flapping frequency, velocity, and acceleration for predetermined specifications of the proposed model in simulations and real-time testing are verified. This comprehensive investigation underscores the efficacy of SRFFC in enhancing the performance and control of FWMAVs. The adaptive fuzzy logic rules employed by SRFFC offer significant advantages in disturbance rejection, control efficiency, and maneuverability, making it a promising control strategy for advanced applications in micro air vehicles. This study compares real-time evaluations of control strategies for the fluttering wing Unmanned Aerial Systems’ leftward, rightward, and forward maneuvers with simulation results. The primary objective of this exhaustive analysis is to further comprehension and refine the design of flapping wing Unmanned Aerial Systems, thereby making a valuable contribution to the advancement of micro air vehicles that are more maneuverable and efficient.

### Variation in wing positions while flapping

**Figure 1:**
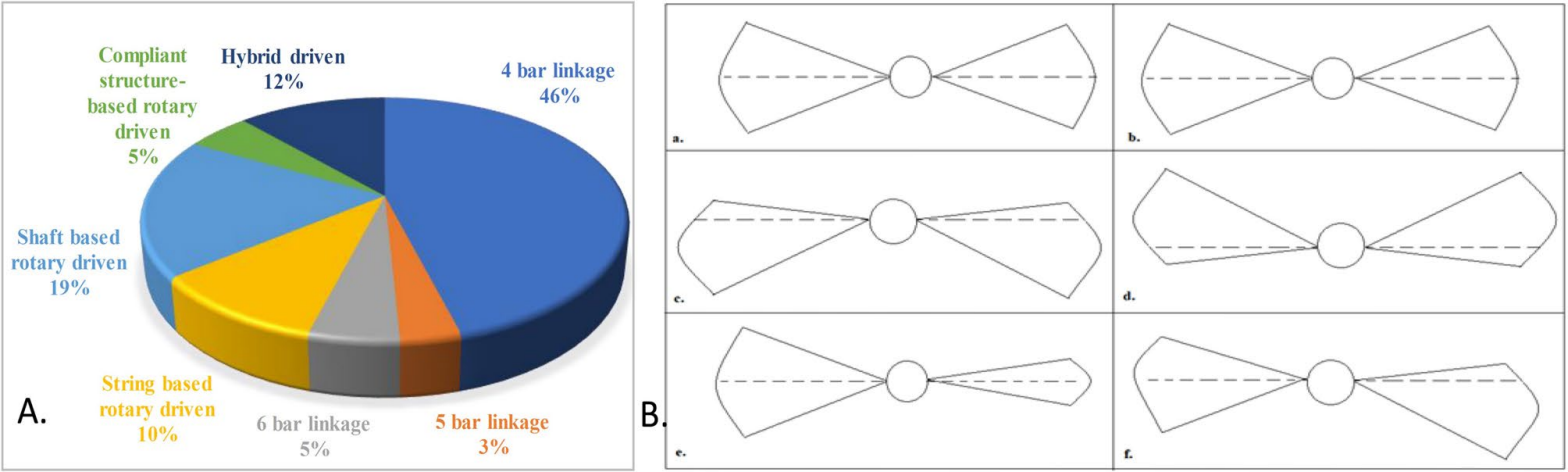
(**A**) Types of Motor driven FWD, depending on flapping actuating mechanisms and (**B**) possibilities of wing movement/positions while flapping with respect to body axis. (i.e. **a** and **b**) Symmetric wing movement. (**c**) and (**d**) symmetric angle of flapping with variations in speed between the wings. (**e**) angle of left wing is greater than right wing. (**f**) asymmetric angle, position, and speed.

Most hovering insects have four distinct wing stroke processes: upstroke and downstroke (translational movement) and pronation and supination (rotational movement). The mean wing speed and lift progressively increase with the increasing flapping frequency and stroke amplitude. However, the mean lift-to-mean wing-tip speed squared ratio decreases if the stroke rate increases, affecting the mean lift coefficient. While the mean lift can increase with the square of the stroke amplitude, vortex shedding precludes the possibility of the mean wing speed increasing. As a result, the more significant flapping frequencies are beneficial since the lift is improvised with no enhancement in the lift-to-torque ratio. The correlation between mean lift and mean drag concerning stroke amplitude is proportional. Expectedly, as the stroke amplitude is varied, the flow mechanics’ mean lift-to-drag and mean lift-to-torque properties have a similar impact^4,30–32^. Based on our literature survey on flapping actuation mechanisms^33–36^, the types of motor-actuated, fully flyable flapping wing (FW) designs identified from Scopus data are illustrated in Fig. 1. The possible wing movements or positions during flapping, relative to the body axis, are shown in Fig. 1B. In Fig. 1B, parts (a) and (b) illustrate symmetric wing movement, while parts (c) and (d) depict symmetric flapping angles with variations in wing speed. Part (e) represents a scenario where the left wing’s flapping angle exceeds that of the right wing, and part (f) demonstrates asymmetric angles, positions, and speeds between the wings. Notably, asymmetric flapping disrupts the synchronized generation of lift forces on both sides, resulting in a reduction in overall lift.

### Button vibrator as (motor) driving source for sliding lever mechanism designs

Vibration is the preferred method for providing feedback in contemporary applications such as touchscreens and gaming controllers. Coin vibrators are typically coreless DC motors, also known as shaftless or pancake vibrators, and are classified as Pico-sized vibration-producing devices. These motors are compact and user-friendly. Until now, they have primarily been used in mobile devices to indicate incoming calls through vibrations. The structural designs of general vibrators can be categorized into three main types: the coin-type, the Eccentric Rotating Mass (ERM/cylindrical) type, and the Linear Resonant Actuator (LRA) type. The differences between these types are summarized in Table 1. Coin vibrators also utilize a spinning offset mass; however, their form factor is flatter and more compact. Unlike cylindrical ERM motors, coin vibrators are completely enclosed, with a short central shaft and an internal flat offset mass, allowing them to maintain their coin-like shape^37–42^. Coin-based vibrators operate on the same principles as ERM motors and can be driven using similar electronic circuits. They require H-bridge circuitry for active braking, and their brushed 3-pole commutation circuit is built around an internal shaft centered on a flat PCB. The rotor consists of a flat plastic disc with a central bearing resting on the shaft, two "voice coils," and a small eccentric mass. These voice coils generate a magnetic field when powered by two brushes on the underside of the plastic disc, which make contact with the PCB commutation pads. This field interacts with the flux produced by a neodymium disc magnet fixed to the motor chassis. The N–S pole pairs embedded in the neodymium magnet are affected by the commutation circuit, which alters the field orientation via the voice coils. As a result, the disc rotates, and the built-in eccentric mass generates asymmetric centripetal forces, leading to net centrifugal displacement, thereby producing vibrations. Button vibrators are commonly integrated into touchscreens or buttons to provide haptic feedback when a user interacts with a device. This feedback enhances user experience by offering tactile confirmation of button presses or touch gestures, making interactions more intuitive^26,37,38,43^.

**Table 1:** Comparison of motor types.

Motor types	ERM types	Coin types	LRA types
Form factor	Cylindrical	Coin	Coin
Working method	Eccentric rotating mass	Eccentric rotating mass	Linear resonant actuator
Built-in connection	Simple/direct	Simple/direct	Uses LRA driver

**Figure 2:**
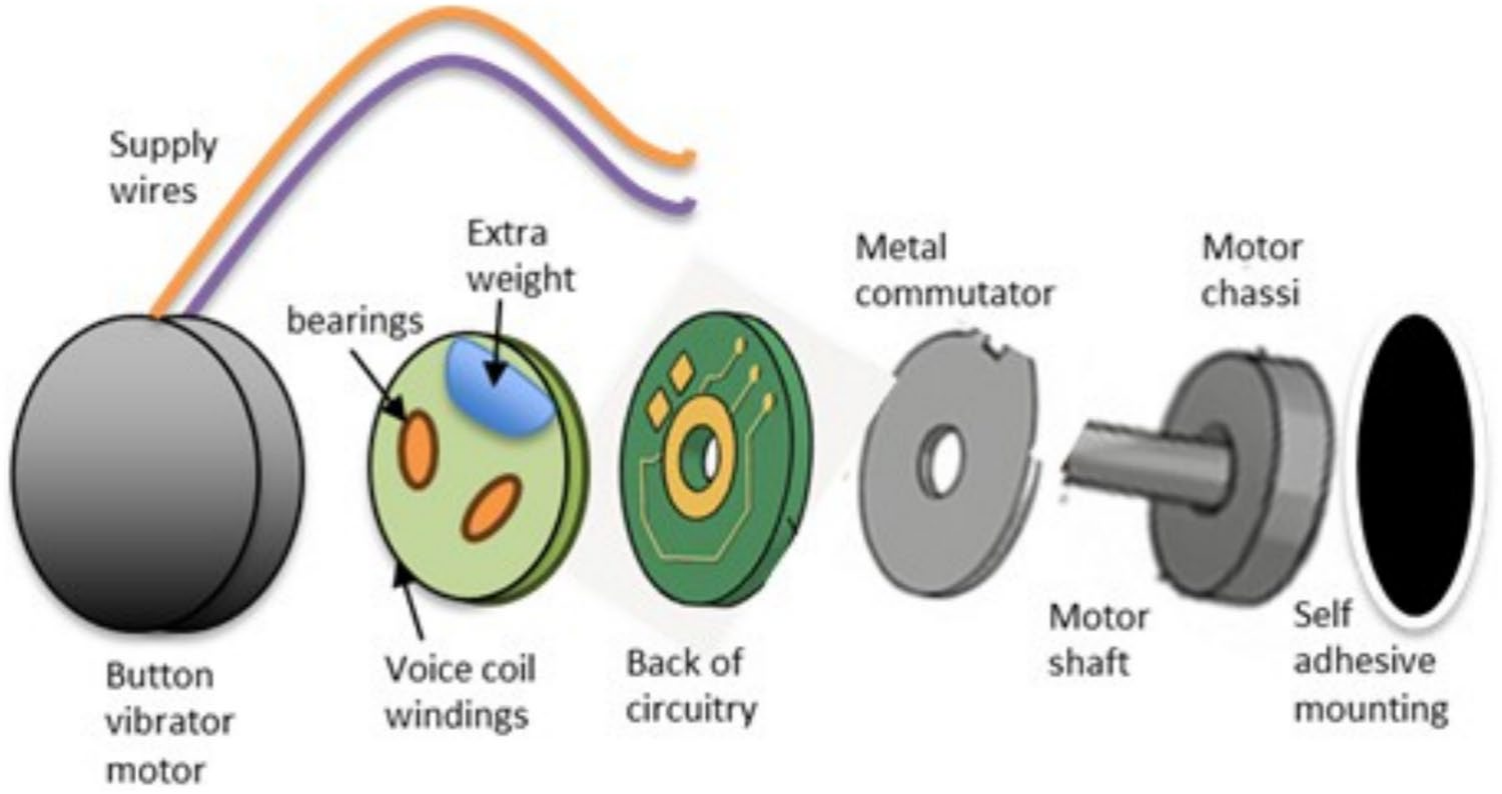
Button vibrator internal structure.

These motors are utilized to generate controlled vibration patterns for capsule motility as it traverses the digestive system of a patient. Capsule endoscopy, a specialized field, requires expertise in electronics, firmware development, and adherence to medical regulations for the development of a comprehensive system^27^. A button vibrator primarily consists of three components: a compact electric motor, an eccentric rotating mass (ERM), and a power source. The motor is typically a small direct current (DC) motor, while the ERM is a small, unbalanced weight affixed to the motor shaft, as depicted in Fig. 2. When an electrical current is applied to the motor, it generates a rotating magnetic field, which interacts with the permanent magnets within the motor to induce shaft rotation. As the motor shaft rotates, the attached eccentric weight also spins, causing an imbalance in the system, which in turn generates vibrations^39,44^. These vibrations are then transmitted to the device or surface on which the motor is mounted. By adjusting the motor’s speed and direction, the intensity and pattern of the vibrations can be modulated to provide a range of tactile feedback effects. To investigate its functionality beyond vibration applications, we disassembled the outer casing of a coin/button vibrator and supplied it with a direct current (DC) power source, operating it as a standard motor. The additional metal mass responsible for generating vibrations was removed, effectively eliminating oscillations. This modified configuration enables the motor to operate with a supply voltage ranging from 1 to 9 V DC, with the rotation speed increasing linearly with the applied voltage. These repurposed coin/button vibrators, now functioning as micro motors, are referred to as coin/button motors in this study. Common FWMAV drive technologies include brushless DC and coreless DC motors coupled via gear-train or slider-crank transmissions^12,22,23,26^, electromagnetic direct drives^34–36^, piezoelectric bimorph actuators, dielectric-elastomer soft actuators, miniature hydraulic or pneumatic drives, and fully mechanical flapping-through- wing-linkage systems^33^. Each offers trade-offs in power density, control precision, bandwidth, and integration complexity. This paragraph cites the comprehensive survey of Singh et al.^33^ Yang et al.^34–36^, and Fenelon and Furukawa^2^ to anchor each category.

## Flapping wing mechanism designs

**Figure 3:**
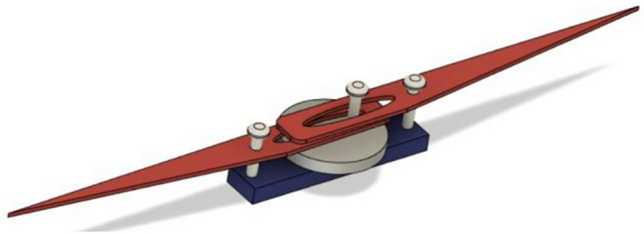
Single Crank-Sliding Dual Lever (SC-SDL) model (DESIGN-1) with single button vibration motor as driving source (The figure is created using Fusion 360 student version software).

The design proposed in this paper is similar to the single crank-slotted dual lever mechanism described in^45,46^ (Crank and Lever Transmission). However, in this study, both the hinges and the motor shaft with the crank are aligned to the origin of the horizontal plane, as illustrated in Fig. 3. The only modification involves replacing the driving motor with a button motor. In electronic devices, button vibrators have traditionally been employed as linear actuators, providing vibration and haptic feedback. However, in this study, we repurpose them as rotating motors for flapping actuation, aiming to develop the smallest possible insect-like structure. The Single Crank-Sliding Dual Lever mechanism design incorporating a single button motor is depicted in Fig. 3. The proposed design has been implemented in CAD, as demonstrated in Fig. 3, where the button vibration motor serves as the driving crank for two opposing levers. The maximum half-stroke wing deflection (flapping angle, *φ*_max_) is determined by the crank-lever geometry and ranges from 15° (Design-1) to 49° (Design-3).

Table 2 collates the primary electrical, mechanical, and aerodynamic parameters for the three prototype drive designs investigated in this work. For each design—SC-SDL (Design-1), SC-SFL (Design-2), and DC-SDL (Design-3)—we list the operating voltage range, attainable flapping frequency, maximum wing-flapping angle (*φ*_max_), wing span, moving mass, input power, and measured thrust. These metrics together illustrate the trade-offs inherent to each actuation strategy: Design-1 achieves a wide frequency sweep at very low mass and moderate thrust, Design-2 produces larger flap amplitudes but over a narrower frequency band, and Design-3 delivers the highest thrust and angle at a fixed nominal frequency. This consolidated overview guides selection of the appropriate mechanism based on specific payload, maneuverability, and energy-efficiency requirements.

**Table 2:** Comparative design and performance specifications of FWMAV actuation mechanisms.

Design	Voltage (V)	Frequency (Hz)	*φ*_max_ (°)	Wing Span (cm)	Mass (g)	Power (W)	Thrust (mN)
SC-SDL (Design-1)	1–5	20–76.3	15	7.5	12.5	0.3–1.2	8
SC-SFL (Design-2)	2–6	5–18	22	8	14	0.4–1.0	12
DC-SDL (Design-3)	3–3.7	30	49	10	15	1.5	25

**Figure 4:**
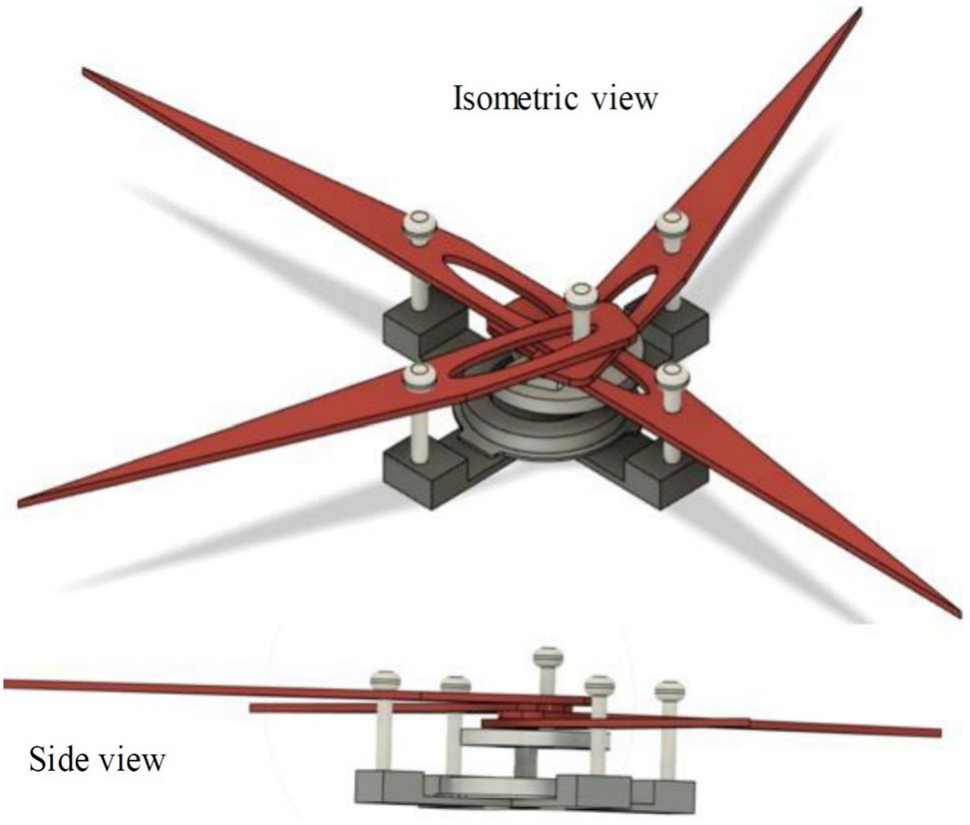
Single Crank-Sliding Quad Lever (SC-SQL) model (DESIGN-2) with single button vibration motor as driving source.(The figure is created using Fusion 360 student version software).

**Figure 5:**
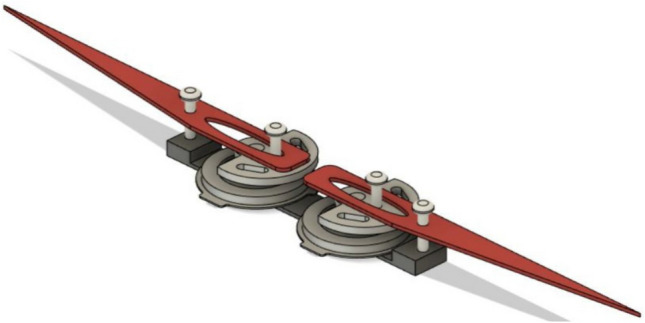
Dual Crank—Slotted Dual Leavers (DC-SDL) model (DESIGN-3) with dual button vibration motor as driving source for individual wing (The figure is created using Fusion 360 student version software).

A single button vibration motor drives the crank, and the crank’s rotation is converted into specific angular motion/flaps by a sliding arrangement at one end of the lever via its slots. The angle of motion produced at its hinge connection is approximately 15–22°, which is slightly lower compared to the flapping angles discussed in the previous chapters. The same design has been modified by adding two additional levers, resulting in slotted quad levers, as shown in Fig. 4. Additionally, it illustrates a side view demonstrating how the levers are stacked one on top of the other to facilitate the smooth movement of all four levers. Figure 5 presents the CAD design of the revised mechanism (Mechanism-III), which is driven by a button motor. This configuration, referred to as the Dual Crank—Slotted Dual Levers (DC-SDL) model, employs dual button vibration motors as the driving source for individual wings.

## Simulation results

This section gives an overview of the results obtained from simulation study.

### Button motor driven design-2 Fwd-model

**Figure 6:**
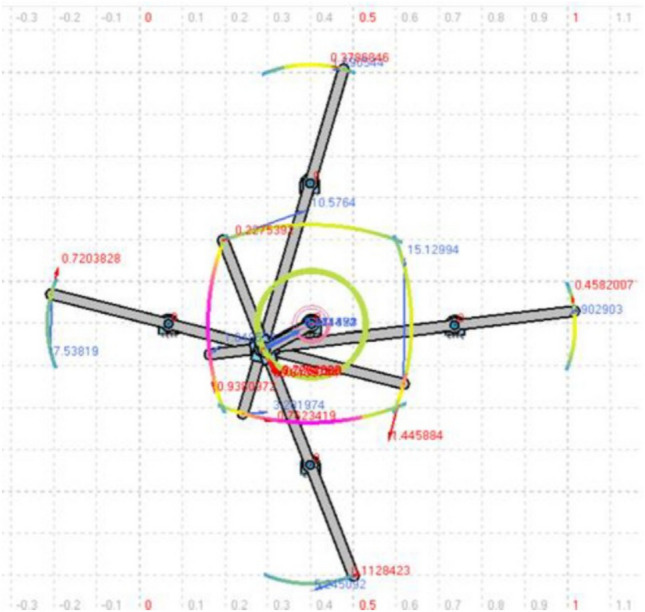
Modelling of four levers (sliding quad levers) actuation through single motor of design-2 in Compmech GIM software with its velocity indications.

**Figure 7:**
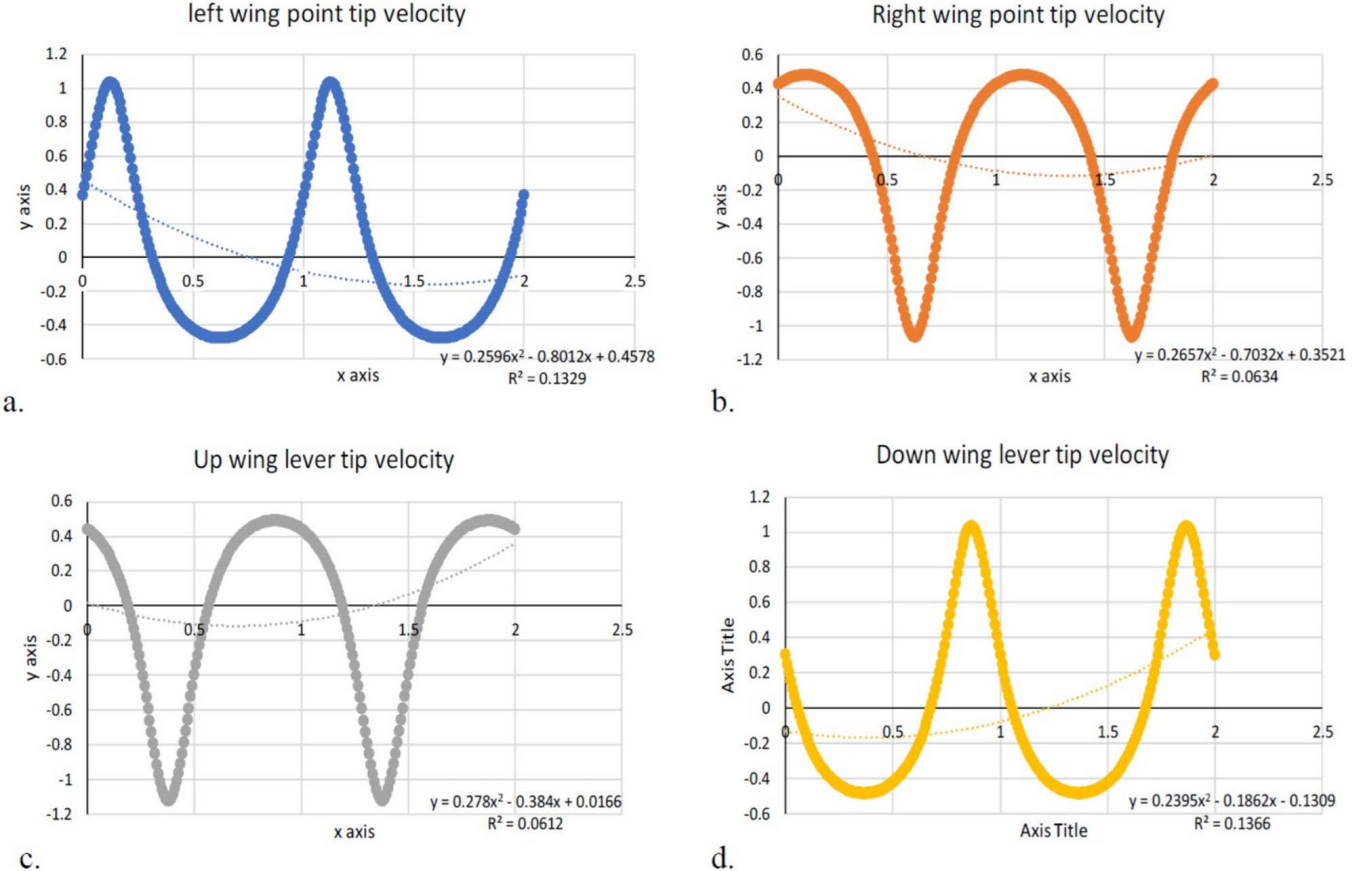
Wing tip velocity variations of all the four (a. left, b. right, c. up, d. down) wings.

**Figure 8:**
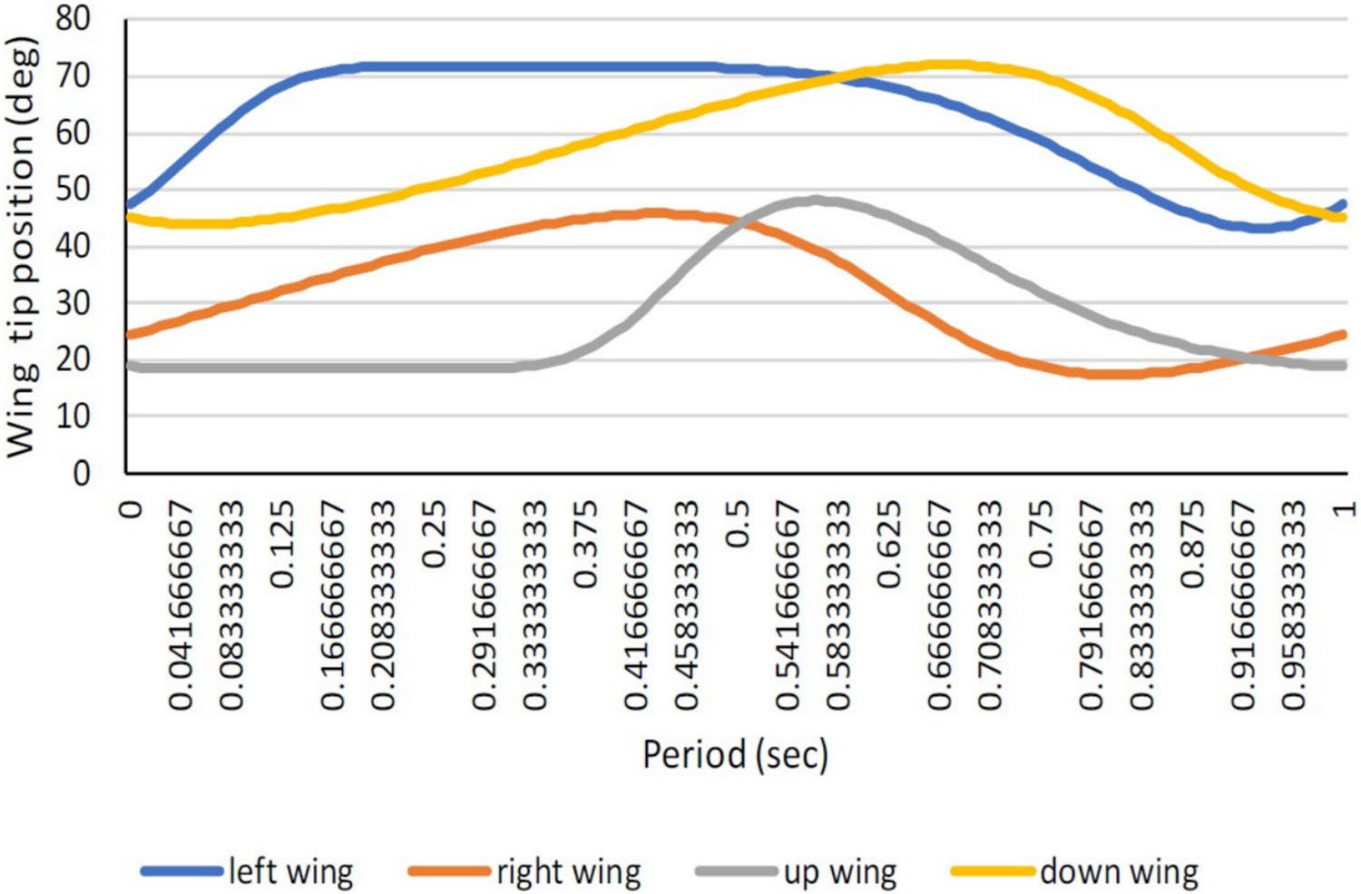
Wing tip positional variations of all the four wings.

**Figure 9:**
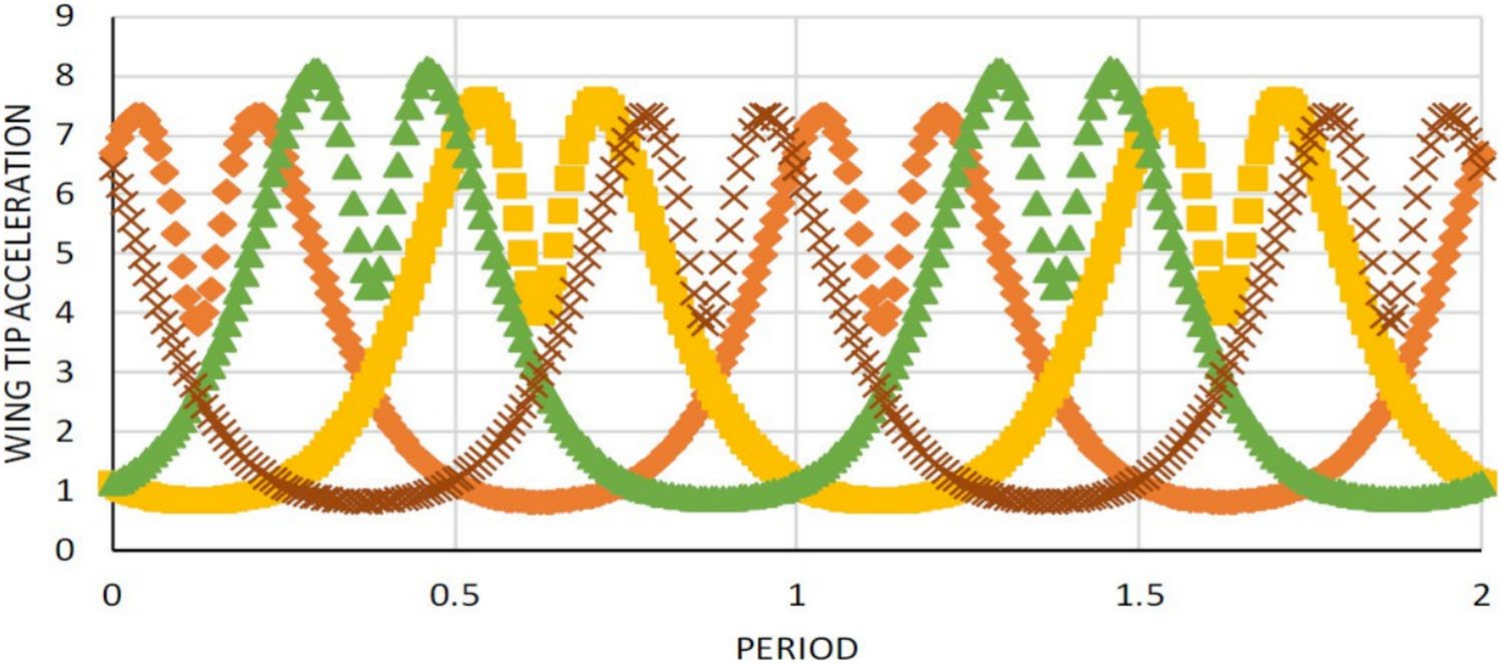
Wing tip acceleration variations of all the four wings.

An attempt was made to add two more wing levers to the same model, and as a result, we were able to achieve the four-lever mechanism that is operated by a single motor, as shown in Fig. 6 of the Compmech GIM software modeling. Modeling of four-wing actuation through a single motor in Compmech GIM software is illustrated in Fig. 6. The obtained wing lever tip angular velocity with respect to the left, right, up, and down levers is shown in Fig. 7. The polynomial curve obtained is highlighted with its equation and *R*^2^ value within the graph. The wing tip positional variations in degrees and angular acceleration for all four levers are depicted in Figs. 8 and 9. The lack of coordination (as seen in Fig. 8) in the lever movements and the resulting insufficient lift can be attributed to various factors such as motor power, torque, uneven load distribution, timely synchronization, mechanical design, and tolerances. Coin motors are typically designed for simple vibration applications and might not have sufficient power or torque to drive a complex mechanical arrangement like a slider-crank mechanism with four levers. As a result, the motor might struggle to overcome the resistance caused by the linkages, leading to uneven movements and reduced lift. In a four-lever arrangement, each lever’s load and resistance might vary depending on the specific geometry of the mechanism. If the linkages and levers are not designed with balanced load distribution in mind, it can result in certain levers receiving more force than others. This imbalance can lead to erratic movements and poor coordination. Achieving proper timing and synchronization of the four levers’ movements is critical for generating consistent and sufficient lift. If the levers are not precisely coordinated, they might interfere with each other’s motion, leading to disruptions in the desired movement pattern. The mechanical design of the linkages, levers, and crank plays a crucial role in the smooth operation of the mechanism. If there are inaccuracies or tolerances in the design, it can result in binding, friction, or misalignment, which in turn can hinder coordination and lift generation. Coin motors often lack the precise control and feedback mechanisms needed for intricate mechanical systems. Without accurate position or speed control, it becomes challenging to ensure that the lever movements are synchronized and coordinated effectively.

**Figure 10:**
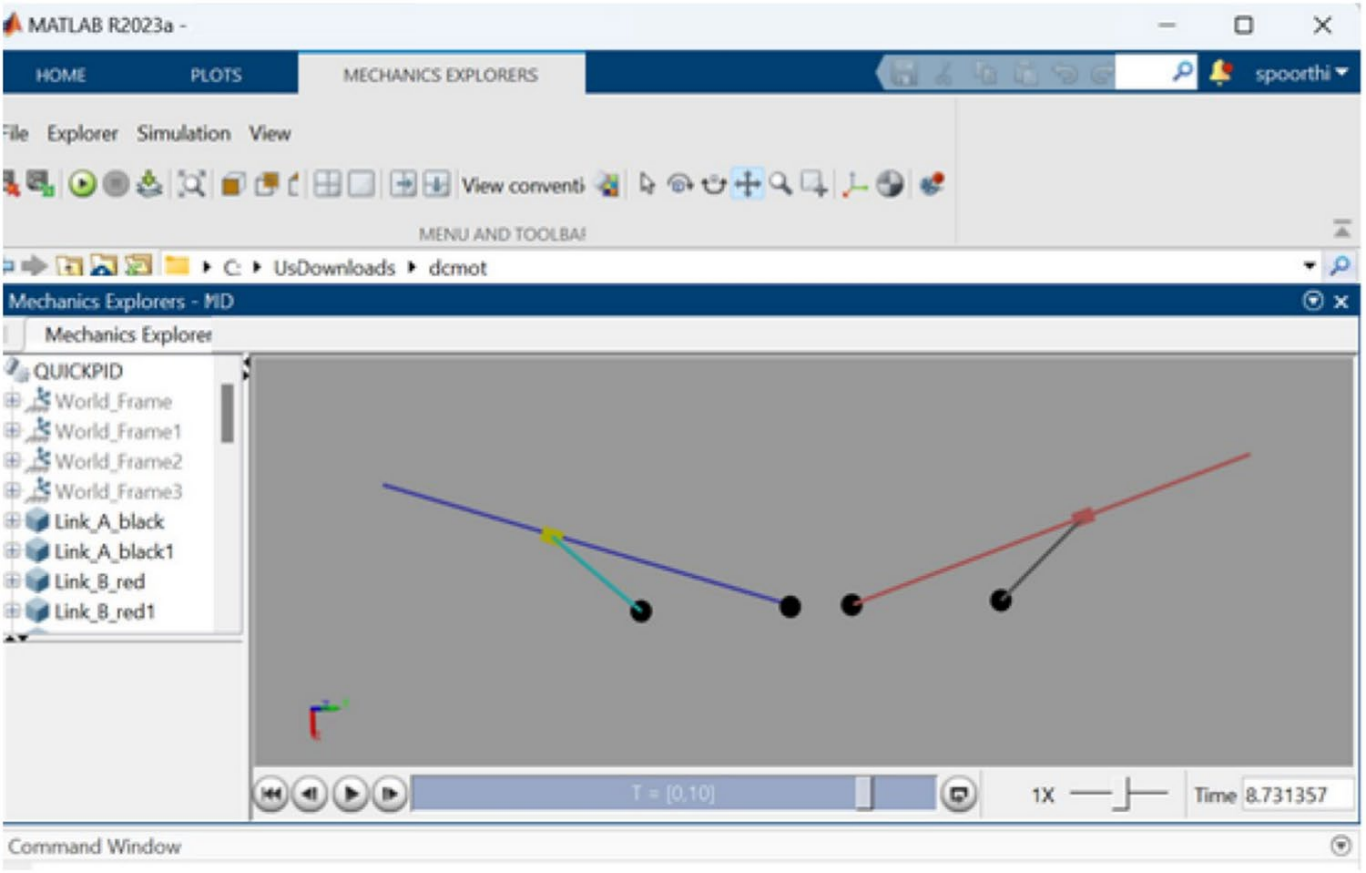
Modelling of Dual Crank—slotted Dual Leavers (DC-SDL) mechanism design-3 in simscape multibody.

**Figure 11:**
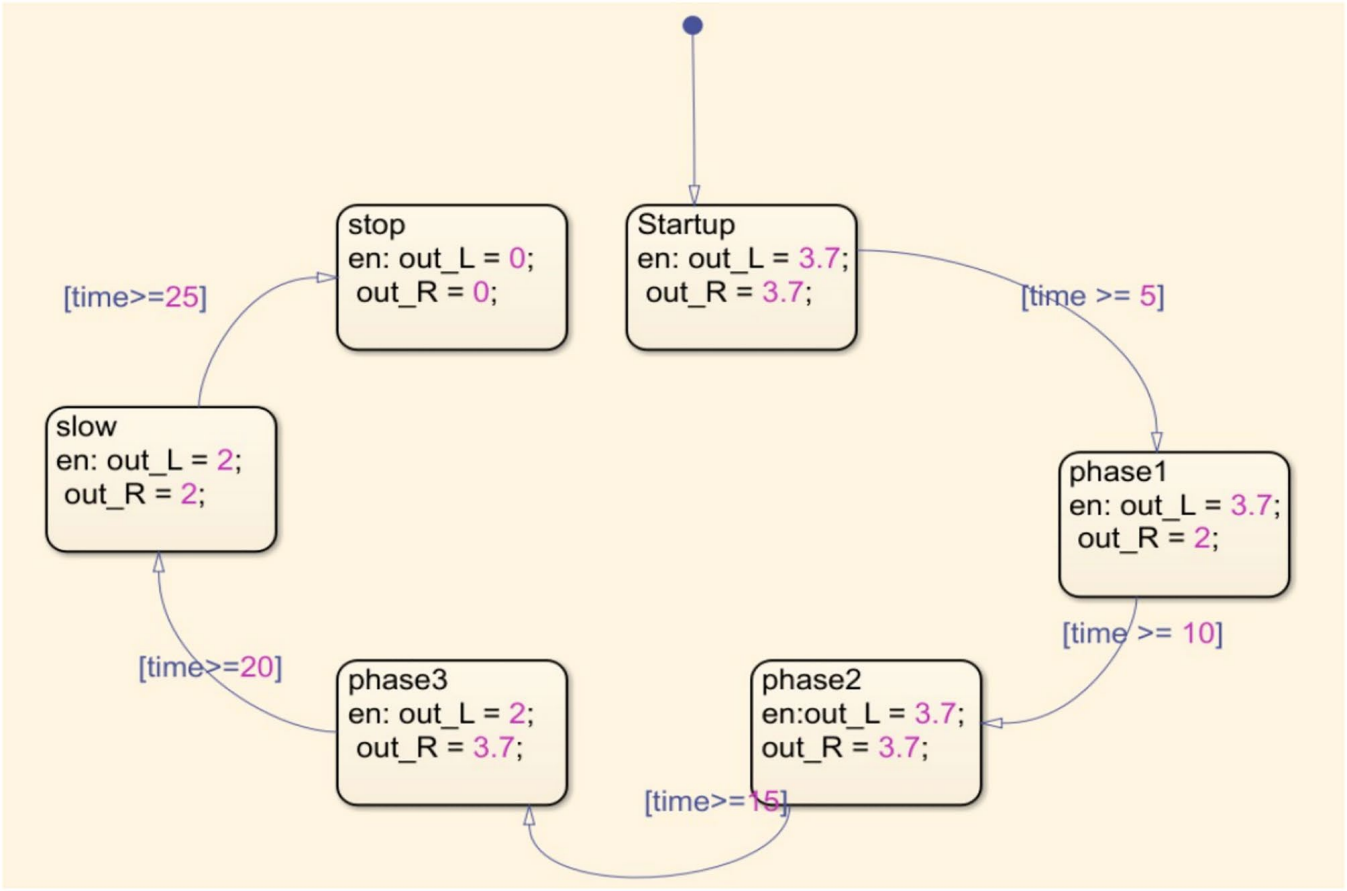
Detailed logic chart for the flapping motion control process.

**Figure 12:**
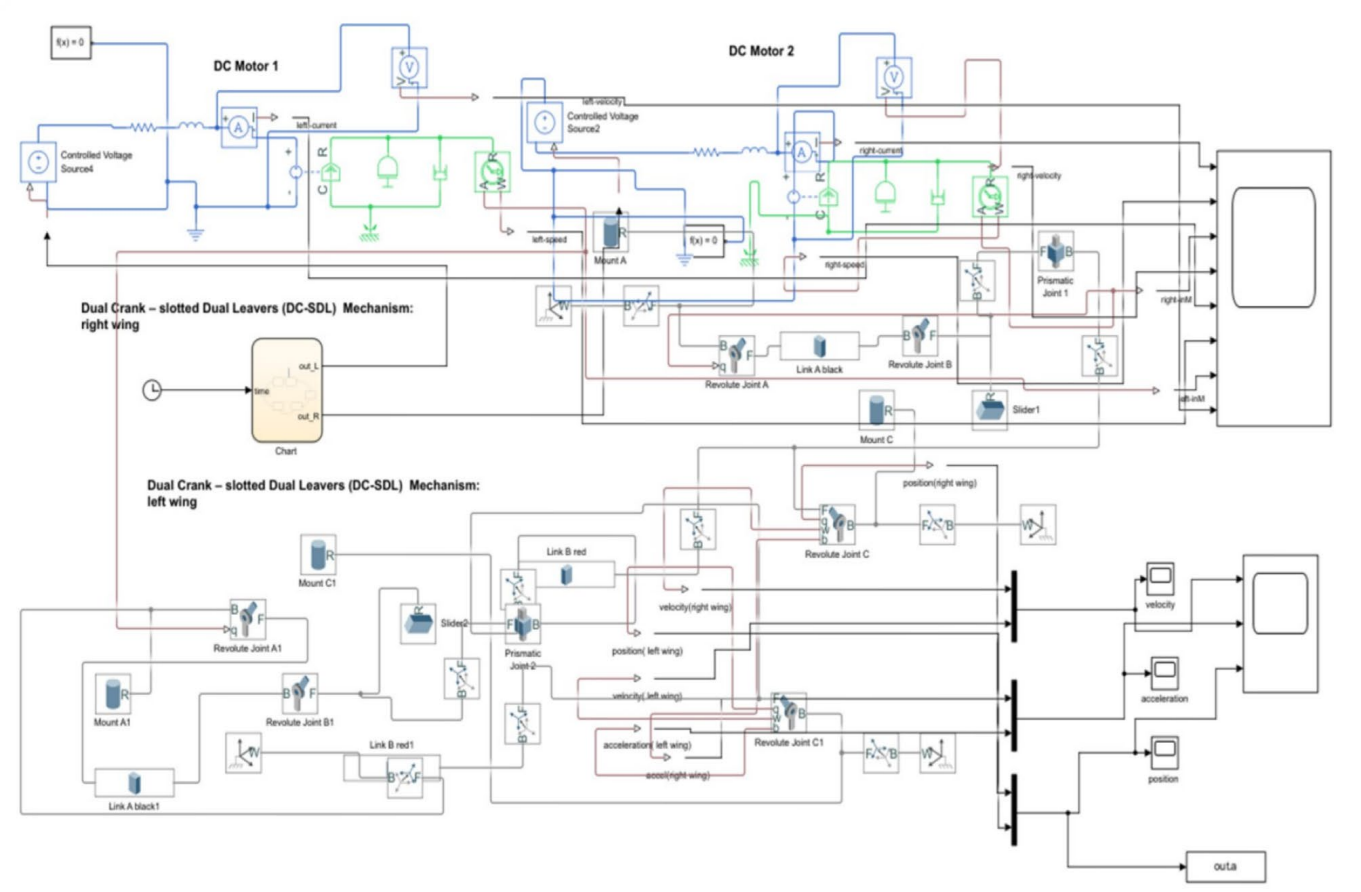
Simscape modelling of Micro-DC motor-controlled DC-SDL mechanism with chart flow process.

Figure 10 presents an illustration of the Simscape Multibody modeling of the DC-SDL mechanism. This modeling demonstrates how different sections have been developed according to the required flow, as depicted in Fig. 11. These sections are represented via blocks and then integrated according to the system requirements. Without establishing the solver configuration for the design, it will not be feasible to actuate the mechanism. Figure 12 provides a visual representation of the detailed joint connections to the blocks that are required for its actuation, along with solver setups. Initially, the single-lever actuation mechanism is constructed, and its movement is validated by actuating it. The left and right lever mechanisms are subsequently developed and activated using two distinct solvers. The input signal is considered as a straightforward signal input for motor joint actuation. As shown in Fig. 10, the blue lever represents the left-side wing connection, while the red lever represents the right-side wing connection. The dummy wing has not been affixed here, as only lever movements are being analyzed, with a focus solely on the mechanism controlling the flaps. Both side levers’ motor-actuated cranks are rotated in opposing directions. Altering the input voltage is necessary to adjust the speed of the flapping wing model (DC-SDL). The logic chart required for this purpose has been constructed in Simscape and is depicted in Fig. 11, where *outL* refers to the voltage control for the left lever and *outR* refers to the voltage control for the right lever. The chart flow indicates that both wings will be beating at the same speed for the first five seconds. After that, the speed of the right wing will fluctuate for the following five seconds, and so on, in accordance with the flow given in Fig. 11.

**Figure 13:**
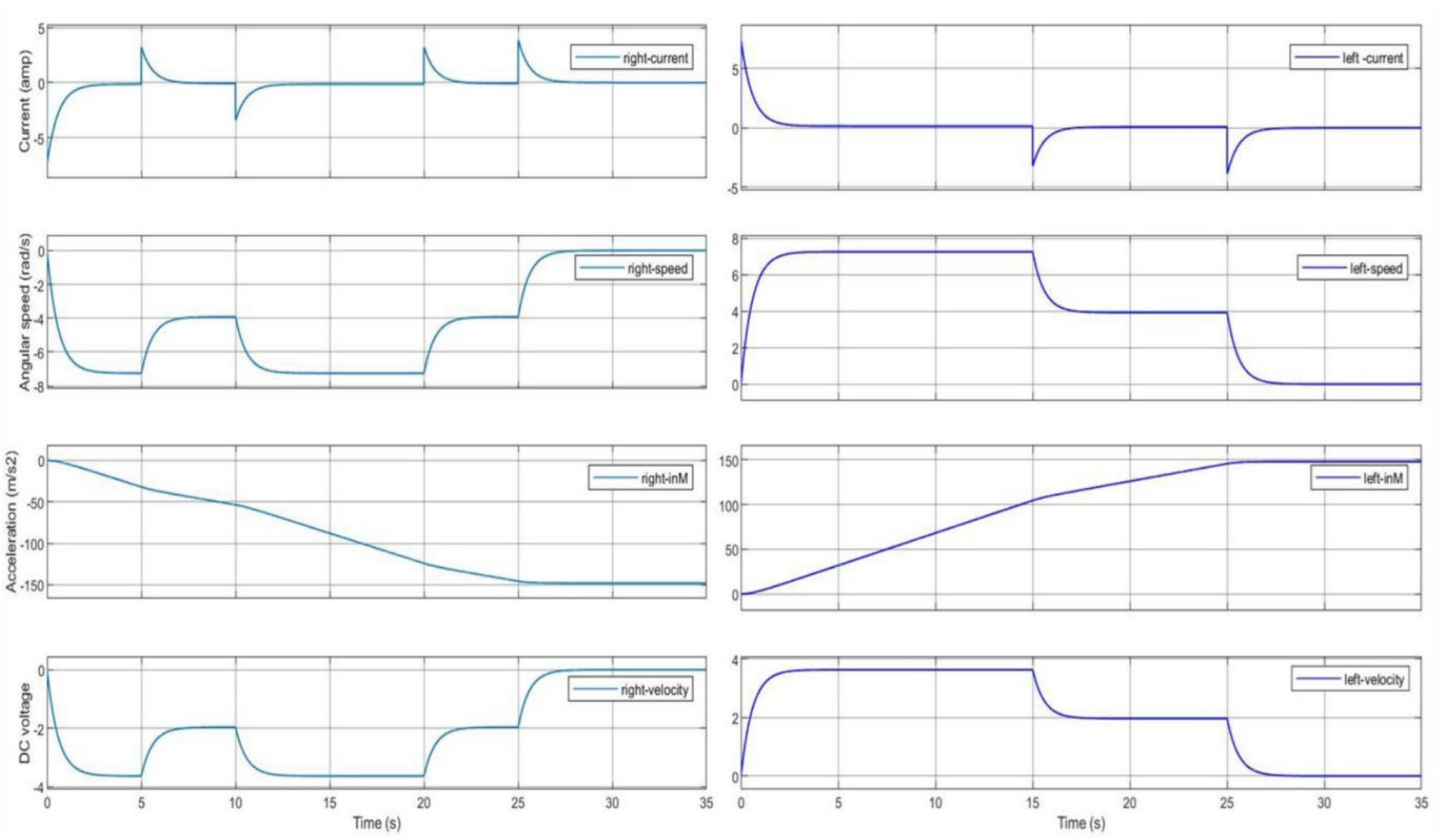
Chart controlled inputs at both motors.

**Figure 14:**
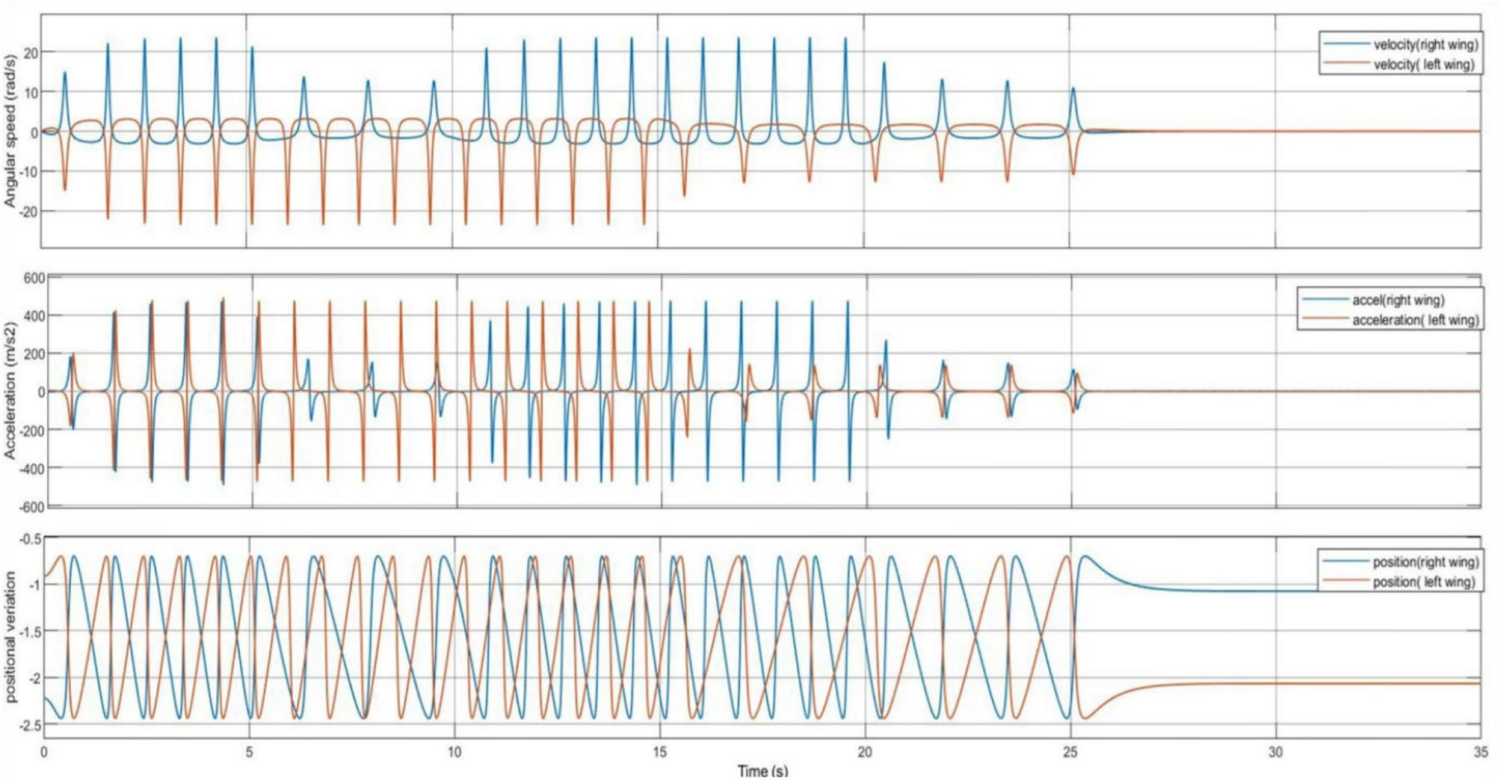
Obtained variations of the flapping lever angular speed, acceleration and position as per the chart flow.

**Figure 15:**
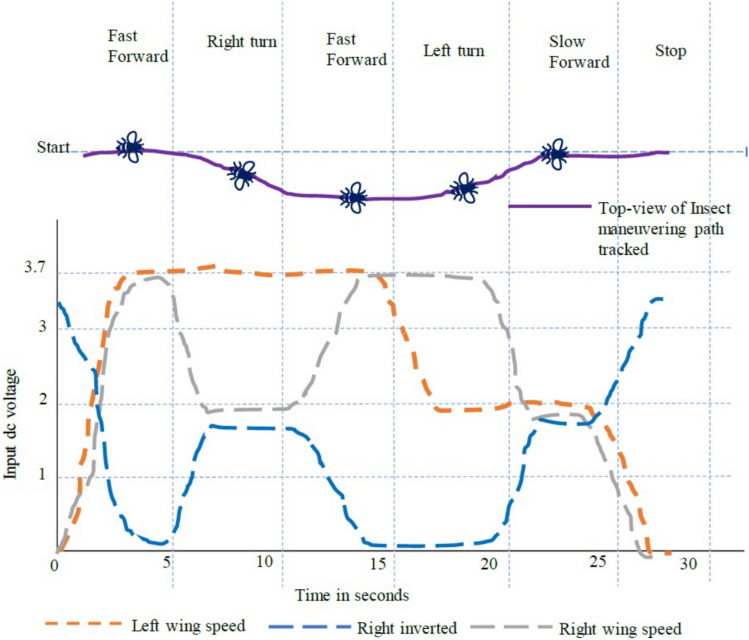
Trace of path for the proposed maneuvering and control with respect to time as per given input dc voltage.

In accordance with the control logic flowchart, the variations in the motor’s DC input voltage and the corresponding changes in motor current, angular velocity, and acceleration are illustrated in Fig. 13. These variations are analyzed for both levers of the DC-SDL model. Figure 14 presents the obtained output, highlighting the lever flapping variations over successive 5-s intervals as per the predefined control sequence. The dynamic response of the flapping lever, including angular velocity, acceleration, and positional variations, is depicted in Fig. 14. During the initial 5-s interval, both wings exhibit synchronized forward motion at an identical velocity. Between 5 and 10 s, an asymmetric control strategy is implemented, wherein the right lever’s velocity is reduced while maintaining a constant velocity on the left lever, inducing a rightward turn. This is followed by a return to symmetric motion in the 10–15-s interval. Subsequently, between 15 and 20 s, the left lever undergoes a velocity reduction while the right lever maintains its speed, generating a leftward turn. In the final 20–25-s interval, both levers operate at a reduced speed of 2 V before coming to a complete stop. These results underscore the critical role of precise input voltage modulation in achieving controlled navigation through independent lever actuation. To validate the maneuverability of the proposed mechanism underground testing conditions, a series of simulations were conducted, as illustrated in Fig. 14. The temporal variations in input voltage applied to the left and right wing actuators are represented in Fig. 15. Given that the dual-motor configuration operates in counter-rotation, the voltage variations of one motor (right) are depicted as inverted, corresponding to the opposite direction of motion. As observed in Fig. 15, coordinated modulation of the left- and right-wing velocities facilitates controlled locomotion. When both motors are driven at 3.7 V, the system exhibits rapid forward motion. A reduction in input voltage to 2 V on either the left or right motor results in a corresponding leftward or rightward turn while maintaining forward motion. When both motors operate at 2 V, the system advances at a reduced velocity, eventually halting upon reducing the input voltage to 0 V.

### Durability and power optimization in motor configurations for FWMAVs

The performance and operational durability of motors in Flapping Wing Micro Aerial Vehicles (FWMAVs) play a crucial role in ensuring reliable, long-duration flight operations. This section presents a comparative analysis of coreless micro motors and button motors, with a focus on novel strategies for enhancing motor durability and optimizing power consumption. Table 3 gives a comparison on various motor specifications and performance matrices.

**Table 3:** Motor specifications and performance (interchanged rows and columns).

	Button motor	Coreless DC motor	Brushed motor
Velocity (m/s)	12.66	12.66	12.66
Lift force (N)	0.10	0.10	0.10
Mech. power (W)	0.1508	0.2827	0.1885
Power consumed (W)	0.50	0.80	0.70
Recovered energy (W)	0.1500	0.2400	0.2100
Efficiency (%)	36.10	52.27	39.85

#### Extended durability testing

**Figure 16:**
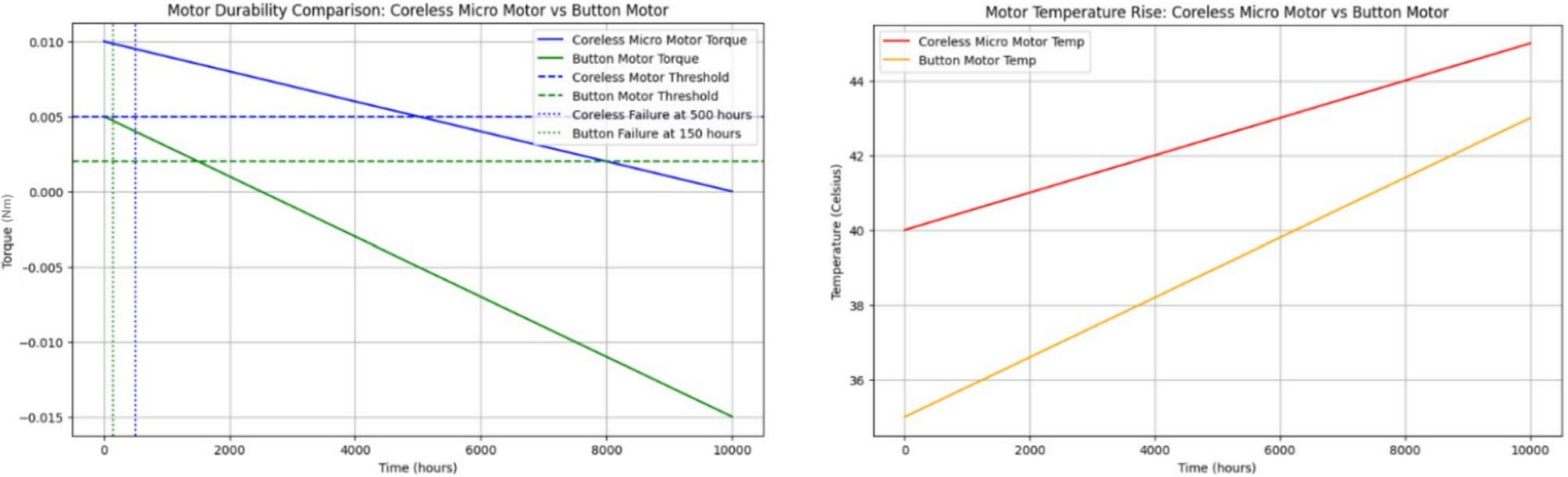
Comparision of motor durability and temperature of both coreless and button motors.

Evaluating motor durability under continuous flapping operations is essential for determining long-term performance. While button motors offer advantages in terms of compactness and lightweight construction, they exhibit accelerated mechanical degradation and increased thermal buildup during extended operation. A comparative assessment of coreless micro motors and button motors, as illustrated in Fig. 16, highlights the superior longevity of coreless motors due to their higher initial torque and lower degradation rate. The key findings from the extended durability tests (referencing Fig. 16) are summarized as follows:

- Coreless micro motors maintain effective torque for up to 7500 operational hours, significantly outperforming button motors, which exhibit a maximum operational lifespan of approximately 3500 h under continuous load conditions.
- Button motors are more susceptible to thermal degradation, experiencing a rapid increase in operational temperature. This accelerated thermal buildup contributes to increased wear, reducing their effective lifespan.

To further analyze motor longevity, an advanced durability modeling approach has been employed to simulate operational stress factors and predict critical failure thresholds. The model incorporates torque reduction and temperature rise over time, enabling a systematic evaluation of motor reliability under real-world operational constraints.

#### Power optimization strategies

Given the limited energy resources in FWMAVs, especially when operating at insect-scale sizes, power optimization is crucial for improving flight duration. In this work, several novel strategies have been applied: i.e. A linear degradation model was applied to track the torque reduction of both button and coreless motors over extended operational hours. A linear temperature rise model was used to simulate the heat accumulation over time. A real-time power management algorithm was used to distribute power based on the motor’s immediate torque and velocity needs.

**Figure 17:**
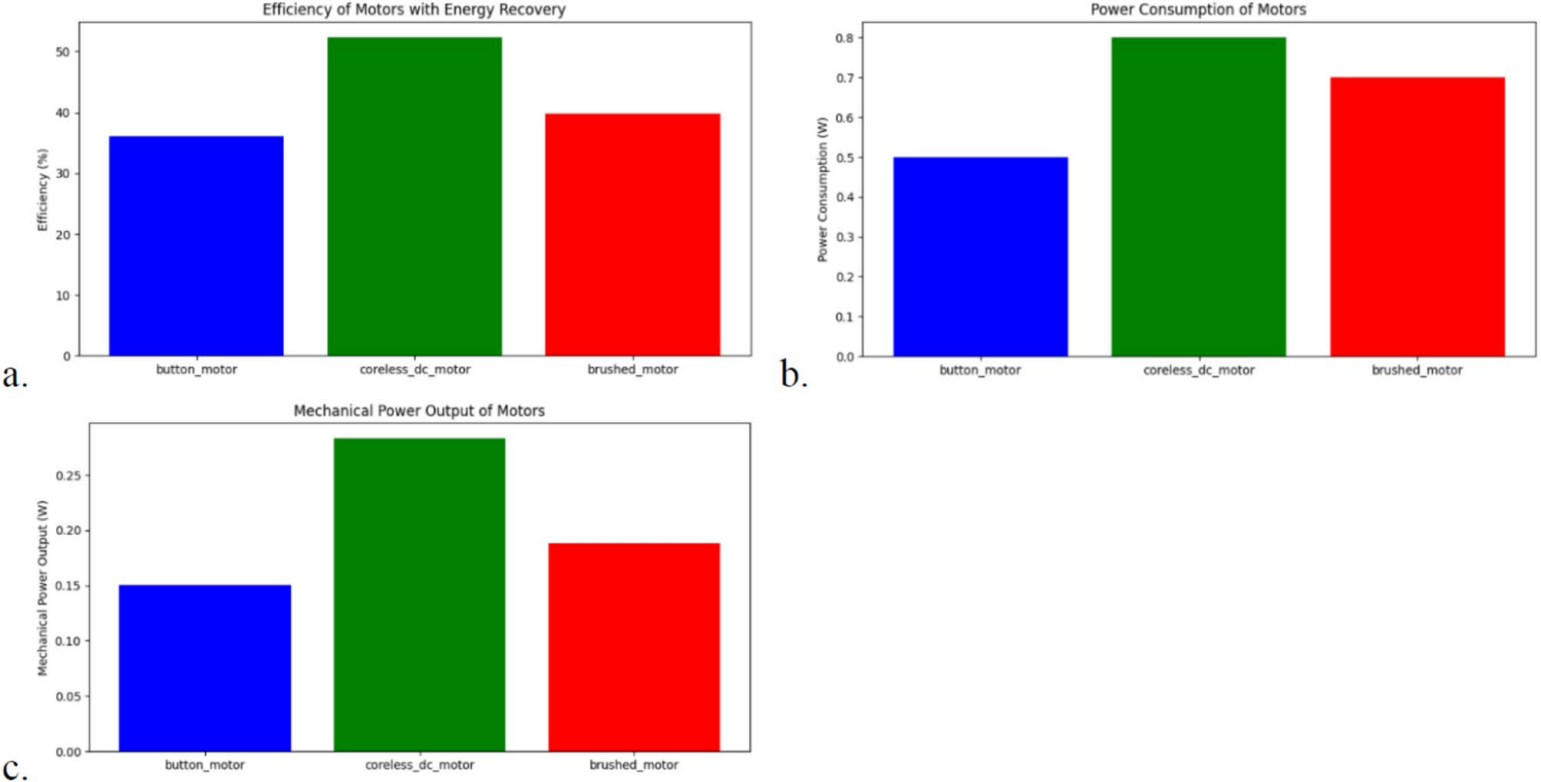
Power optimization, efficiency and modular motor configurations.

- Energy recovery during wing downstrokes, where a portion of the energy consumed during the downstroke of the wing is recovered and feed back into the system. For instance, a recovery factor of 30 pec can significantly improve energy efficiency (Fig. 17).
- Optimized power distribution algorithms, which adjust the power sent to motors based on real-time torque requirements, allowing the system to minimize unnecessary energy expenditure. Simulation results show that the coreless DC motors, with their higher torque output and better mechanical efficiency, benefit most from these power optimization strategies, reaching an efficiency with energy recovery of 52.27 pec, compared to 36.10 pec for button motors (Fig. 17).

#### Modular motor configurations

To enhance manoeuvrability and control, modular motor configurations were explored. By driving each wing independently with a dedicated motor, the FWMAV can achieve improved directional control and robustness in the case of motor failure. This approach not only improves control precision but also allows for redundancy in design, which can be critical in search-and-rescue applications where reliability is paramount. The results of this study suggest that while button motors offer advantages in weight and size, their application is more suited to short-term operations or where lower power consumption is prioritized over long-term durability. In contrast, coreless motors, though slightly heavier, provide better performance for longer-duration flights and more demanding missions. These strategies discussed above, ranging from durability testing and temperature control to energy optimization and modular motor configurations—work synergistically to enhance the performance, efficiency, and reliability of FWMAVs. The introduction of energy recovery and independent motor control provides a marked improvement over traditional systems, offering longer flight times, greater control precision, and improved durability.

### Simulation results of SRFFC and FPID

Both the Self-Regulatory Fractional Fuzzy Control (SRFFC) and Fractional PID (FPID) controllers are advanced control strategies designed to effectively handle nonlinearities and external disturbances in Flapping Wing Dynamics (FWD). In the context of Micro Aerial Vehicles (MAVs), where precise wing motion control is critical, these strategies offer significant advantages in maintaining stability and responsiveness. A comprehensive analysis of key performance metrics—including rise time, settling time, overshoot, and the Integral of Absolute Error (IAE)—is essential for selecting the most suitable controller to achieve the desired performance characteristics in MAV applications. The SRFFC controller leverages fuzzy logic to dynamically adjust its parameters based on the error, its rate of change, and historical performance data. This adaptive tuning mechanism enhances system response under varying operational conditions, improving robustness and adaptability compared to conventional fixed-gain controllers.

1 $$U_{SRFFC} = K_{p} e + K_{i} \smallint edt + K_{d} \frac{de}{{dt}}$$

where *e* is the error between the desired and actual positions. *K*_*p*_, *K*_*i*_, and *K*_*d*_ are the proportional, integral, and derivative gains, respectively. These gains are adaptively tuned based on error values and are influenced by fuzzy logic rules. The Fractional PID (FPID) Controller introduces fractional calculus into the PID framework, providing a more flexible control response that can be fine-tuned for systems with complex dynamics. This approach uses fractional powers to adjust the influence of integrative and derivative terms, allowing more nuanced tuning of the controller’s response.

2 $$U_{SRFFC} = K_{p} e + K_{i} \smallint edt\,\alpha + K_{d} \frac{de}{{dt}}\,\beta$$

*α* and *β* are the fractional orders of the integral and derivative actions, respectively.

As illustrated in Fig. 15, the maneuvering of the Flapping Wing Dynamics (FWD) system using a button motor or DC motor is simulated at 5-s intervals. The simulation employs both Self-Regulatory Fractional Fuzzy Control (SRFFC) and Fractional PID (FPID) controllers to analyze their effectiveness in regulating motor dynamics. The motor dynamics function models two motors, denoted by *θ*_1_, *ω*_1_, *i*_1_ and *θ*_2_, *ω*_2_, i_2_, which rotate in opposite directions. The SRFFC strategy employs a self-regulating fractional fuzzy control mechanism, which dynamically adjusts control parameters based on error dynamics, ensuring adaptive performance. In contrast, the FPID controller incorporates fractional integral and derivative actions to enhance stability and tracking accuracy. In the implemented Python program, the control voltage function assigns voltage levels to both motors, varying every 5 s to simulate speed variations as specified. The desired position function defines target positions for each motor at every 5-s interval, corresponding to the voltage changes. The system dynamics are computed using the odeint solver, which numerically integrates the system’s ordinary differential equations for both control strategies. To maintain practical feasibility, control inputs are constrained within a predefined range to prevent excessive voltage variations.

**Figure 18:**
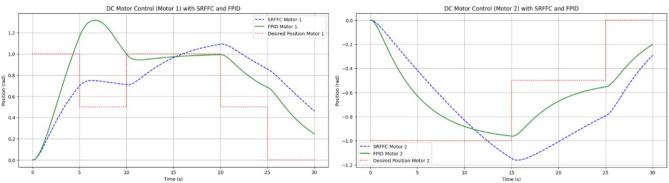
Trace of path for the proposed maneuvering and control of button/DC motors with SRFF and FPID.

The plot shown in Fig. 18 compares the responses of SRFFC and FPID controllers for each motor against the desired positions over time. Dashed lines represent the desired positions, providing a reference to evaluate tracking accuracy. These plots help visualize the control strategy that offers superior tracking performance and stability under varying conditions. From the results in Fig. 18, key performance metrics such as response time, overshoot, and steady-state error for each controller can be analyzed, offering insights into their respective advantages. This simulation provides a comprehensive comparison between SRFFC and FPID controllers for two motors with varying speed inputs, demonstrating their effectiveness in motion control. Practical systems often encounter unexpected disturbances, which can cause deviations from the desired system output. Efficient control strategies must effectively reject such disturbances to ensure accurate tracking. In this study, disturbances are applied to both motors, as illustrated in Fig. 20, to evaluate the controllers’ ability to mitigate these effects. Figure 20 presents the applied disturbance profiles along with the response of both motors under SRFFC and FPID control strategies, highlighting their resilience against disturbances. To enhance disturbance rejection, a neuro-fuzzy disturbance observer observer is implemented as a simple AI-based model that learns the effect of disturbances on the system by continuously analyzing the error dynamics. The disturbance observer employs a self-tuning fuzzy-inference system with Gaussian membership functions. Its rule weights and membership parameters are continuously updated via a gradient-descent learning law, enabling real-time adaptation to changing aerodynamic loads. This neuro-fuzzy structure constitutes the machine-learning component of our control scheme .The observer estimates external disturbances based on the deviation between the actual and desired system outputs and compensates for them to maintain stability and tracking accuracy. The estimated disturbance is computed as:

3 $$\hat{d}(t) = \gamma (y_{d} - y_{a} ) + \hat{d}(t - {1})$$

where $\hat{d}$(*t*) is the estimated disturbance, *y*_*d*_ is the desired output, *y*_*a*_ is the actual output, and *γ* is the observer gain, which is a tuning parameter dictating the sensitivity and reaction speed of the observer. The observer modifies the control input accordingly to counteract the disturbance’s effect. By integrating SRFFC and FPID control strategies with a disturbance observer, the system’s ability to reject disturbances and maintain precise tracking is significantly improved. Adaptive control techniques and AI-based estimation enhance the system’s robustness, ensuring reliable operation under unpredictable conditions.

**Figure 19:**
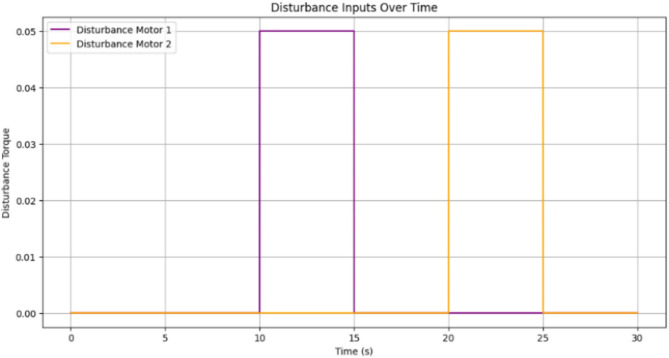
Disturbances inputs over time, for two motors.

**Figure 20:**
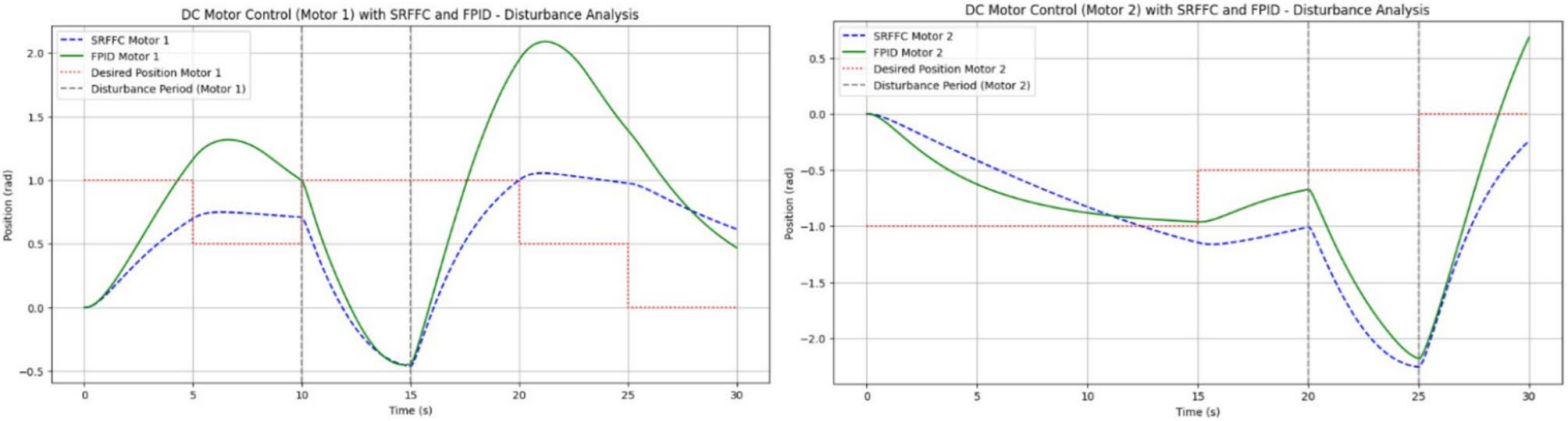
Trace path of disturbance analysis with desired positions of both motors with SRFF and FPID.

**Figure 21:**
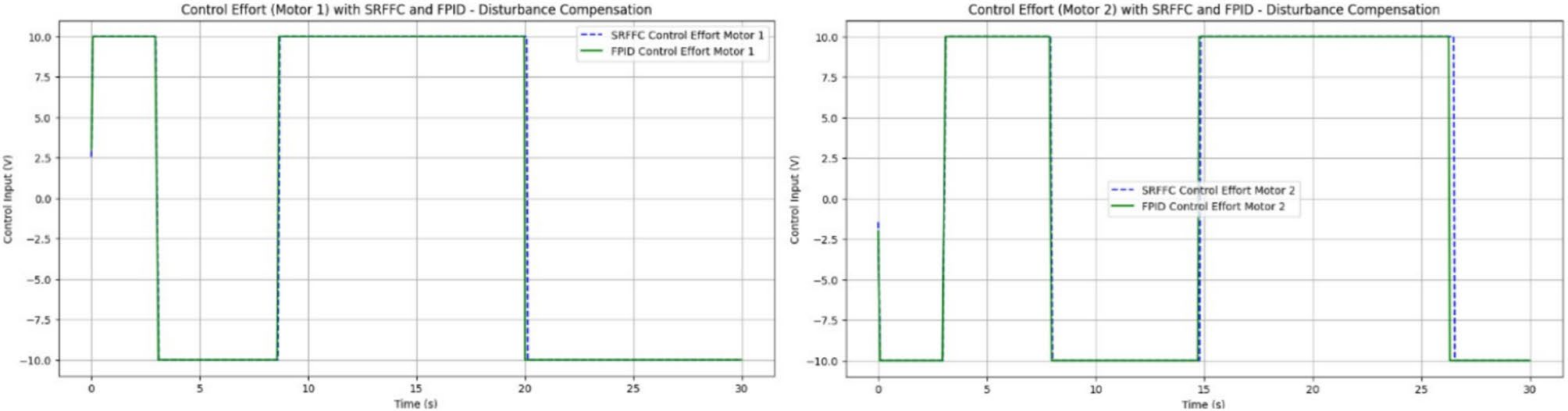
Control effort to both motors for Disturbance compensation.

Figures 19 and 20 illustrate the difference between actual and desired positions over time. A smaller tracking error indicates better performance. Figure 20 further depicts the disturbance torque applied to each motor, allowing for a correlation between system response and disturbance periods. Meanwhile, Fig. 21 presents the control input (effort) applied by each controller to maintain the desired position, reflecting the energy required to achieve the tracking objective.

Performance metrics derived from Fig. 20 are summarized in Table 4. These include:

- *Rise Time*: The time interval between the system response reaching 10% and 90% of the final desired value.
- *Settling Time*: The time required for the response to remain within 2% of the final desired value.
- *Overshoot*: The maximum deviation from the final desired value, expressed as a percentage.
- *Integral Absolute Error (IAE)*: Computed using the trapezoidal rule to integrate the absolute error over time.

**Table 4:** Performance comparison between SRFFC and FPID Controllers: simulation output regarding rise time, settling time, overshoot and IAE (without any disturbance).

Motor	Controller	Rise time (s)	Settling time (s)	IAE
Motor 1	SRFFC	0.00	0.00	56.41
Motor 1	FPID	0.00	0.00	55.77
Motor 2	SRFFC	0.00	0.00	52.53
Motor 2	FPID	0.00	0.00	51.81

**Figure 22:**
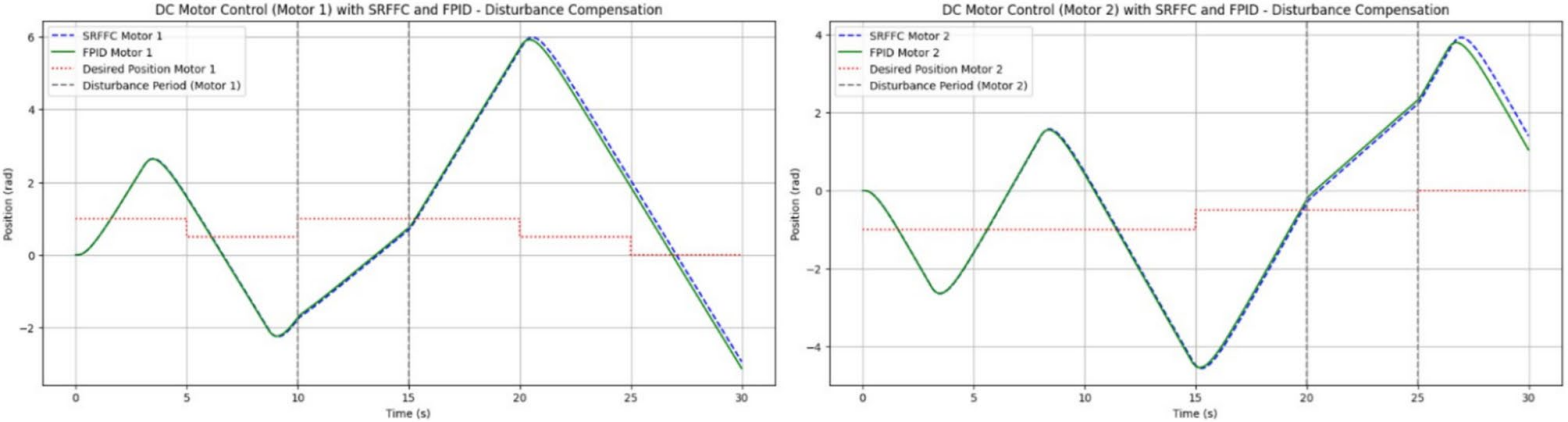
Disturbance compensation with desired positions trace path for both motors with SRFF and FPID.

**Figure 23:**
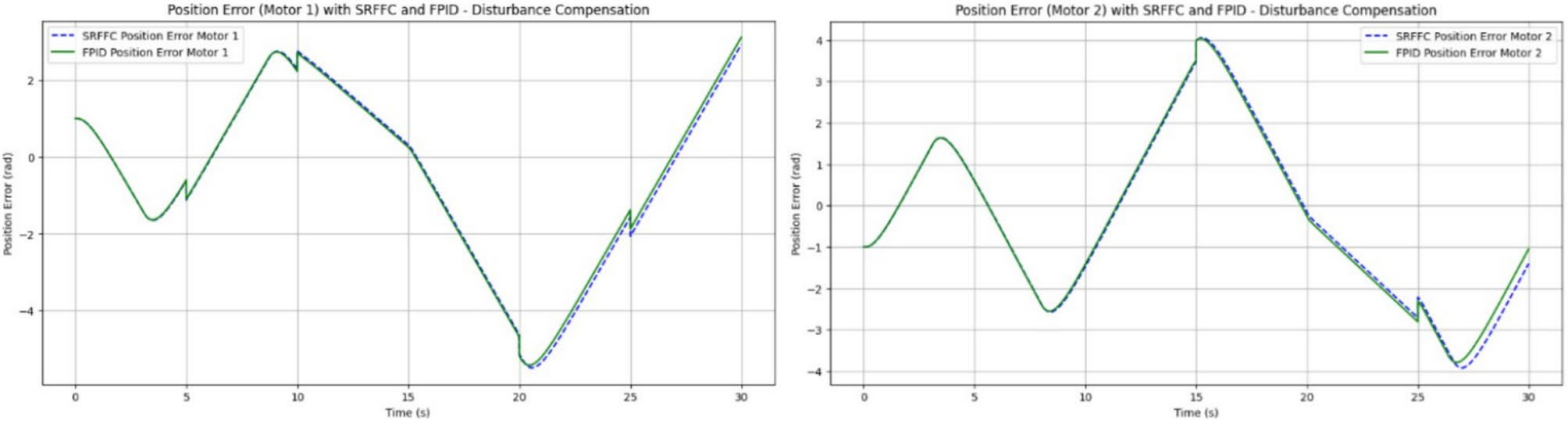
Position error with desired positions trace path for both motors.

From Table 4 the Motor 1: SRFFC underperforms FPID by approximately 1.15 pec in disturbance rejection (lower IAE is better). Motor 2: SRFFC underperforms FPID by approximately 1.39 pec in disturbance rejection. This suggests that FPID demonstrates marginally better performance in this specific metric of disturbance rejection. Both controllers are equipped with disturbance observers, which can estimate and compensate for external disturbances as shown in Fig. 22. This is particularly useful in MAV applications where aerodynamic disturbances are common. The disturbance observer ensures that the system remains stable and performs well even under varying external conditions. The responsiveness (rise time) and precision (settling time and overshoot) are critical for MAVs to maintain stable and efficient flight. The above simulations provide a way to analyse the metrics, through this by fine-tuning the control parameters to achieve the desired flapping wing motion will be achievable. In a real MAV, there are physical constraints and limitations such as motor saturation, mechanical wear and tear, and power supply limitations. These factors should be considered when implementing the control strategies. Figure 23 indicates the position error after disturbance compensation, with desired positions trace path for both motors. Because the position error is very minimal, it can be neglected.

**Figure 24:**
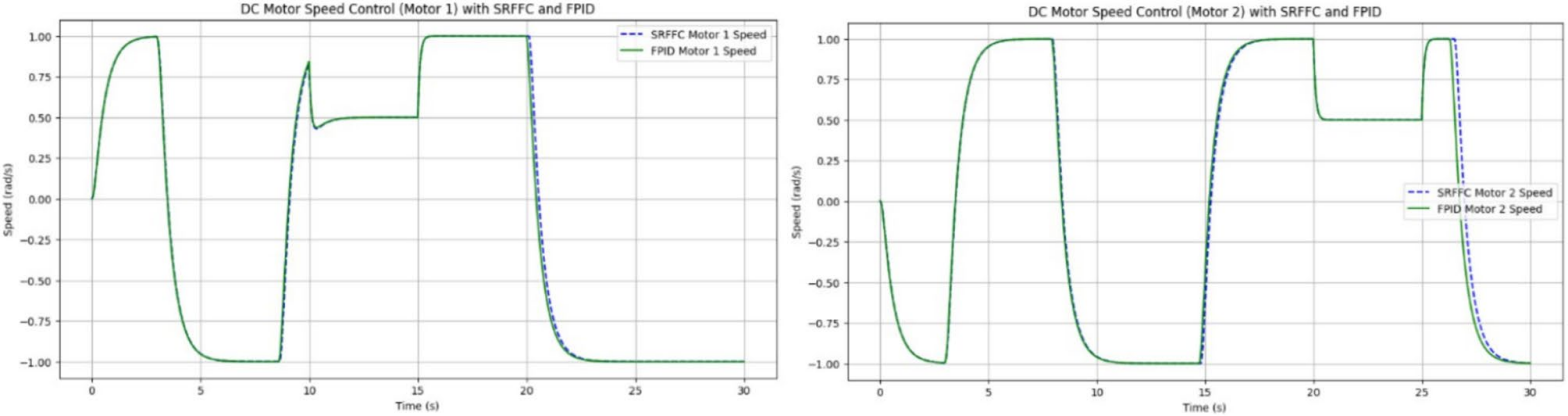
Trace path of Speed analysis with desired positions of both motors with SRFF and FPID.

SRFFC Motor 1 Speed as in Fig. 24, (blue, dashed line) in its Initial Phase (0–5 s), The speed increases rapidly, indicating a quick response to the control input. This is due to the high voltage applied initially during the disturbance Period (10–15 s), There is a noticeable dip in speed, indicating the disturbance torque affects the motor. However, the controller compensates and brings the speed back to the desired trajectory. While Final Phase (25–30 s): The speed gradually reduces as the control voltage decreases, leading to a smooth approach to zero.

FPID Motor 1 Speed as in Fig. 24 (Green, solid line), in its Initial Phase (0–5 s), Similar to the SRFFC, the FPID controller also results in a rapid increase in speed, showing effective initial control. During disturbance period (10–15 s), the speed drop is more pronounced compared to SRFFC, indicating that FPID might be slightly less effective in handling the disturbance. During its Final Phase (25–30 s), the speed decreases, but with slightly more oscillations compared to SRFFC, indicating a less smooth transition to the final value. SRFFC Motor 2 Speed as in Fig. 24 (Blue, dashed line), in its Initial Phase (0–5 s), The speed profile shows a quick response similar to Motor 1, reflecting the initial control effectiveness. While Disturbance Period (20–25 s): The speed shows a clear dip due to the disturbance, but the controller compensates effectively, bringing the speed back to the desired trajectory. At its Final Phase (25–30 s): The speed reduces smoothly, approaching zero without significant oscillations. FPID Motor 2 Speed as in Fig. 22 (Green, solid line), in its Initial Phase (0–5 s): The speed increases rapidly, similar to Motor 1, showing effective initial control. During the Disturbance Period (20–25 s): The disturbance effect is more pronounced with a sharper dip in speed, indicating FPID’s slightly lower disturbance rejection capability. At its Final Phase (25–30 s): The speed decreases with more oscillations compared to SRFFC, indicating a less smooth transition to the final value.

**Figure 25:**
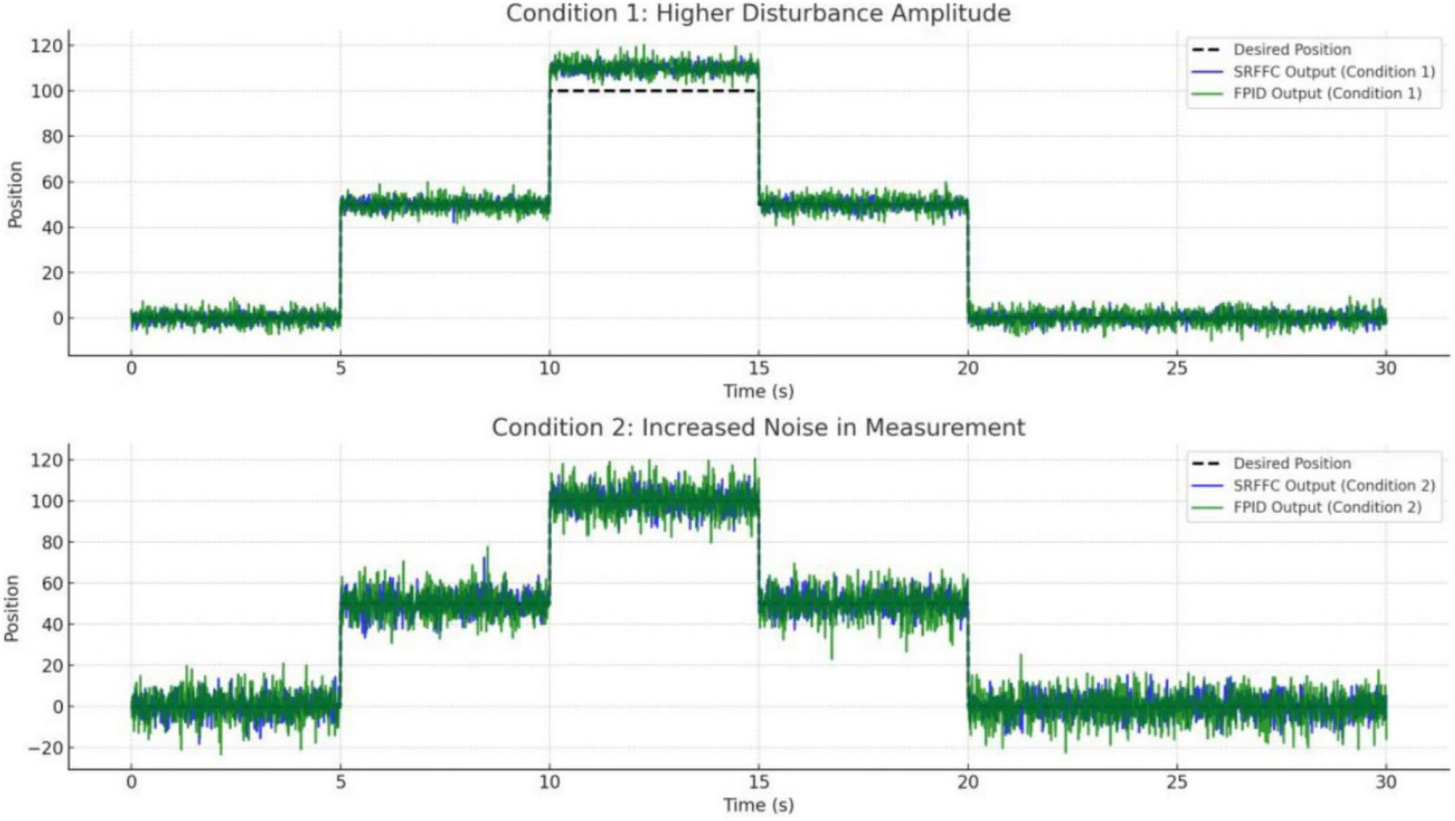
System’s performance under two different conditions.

From the above simulation analysis, both SRFFC and FPID controllers exhibit quick rise times, demonstrating their effectiveness in rapidly bringing the motors up to speed. However, a comparative evaluation of key performance metrics reveals notable differences in their handling of disturbances and overall stability. The SRFFC controller exhibits superior disturbance handling, showing smaller transient deviations and faster recovery compared to FPID. The settling behavior of SRFFC is smoother, with fewer oscillations in the final phase, indicating improved overall stability. Overshoot is more pronounced in the FPID controller, particularly during disturbance periods, suggesting that SRFFC provides better control in mitigating excessive deviations. The SRFFC controller maintains a more stable speed response with reduced oscillations, particularly towards the end of the simulation. While both controllers effectively achieve desired speeds, SRFFC outperforms FPID in mitigating overshoot and maintaining steady-state stability. This enhanced stability makes SRFFC a more robust choice for applications requiring precise and stable motor control, particularly in the presence of external disturbances. From Fig. 25, the Condition 1 (Higher Disturbance Amplitude): The SRFFC controller demonstrates superior disturbance rejection, with lower overshoot and faster settling time compared to FPID, highlighting its adaptability in aggressive environments. From Fig. 25, the Condition 2 (Increased Measurement Noise): The SRFFC controller achieves smoother tracking with fewer oscillations, indicating superior robustness to sensor noise. In contrast, the FPID response exhibits higher fluctuations and error accumulation, making it less reliable in noisy conditions. These findings, illustrated in Fig. 25, reinforce the effectiveness of SRFFC for applications requiring precise control under challenging conditions, such as flapping-wing drones. The superior adaptability and disturbance rejection of SRFFC position it as a promising candidate for further exploration in micro air vehicles (MAVs). Conversely, while FPID remains a flexible control strategy, its lower robustness in highly dynamic or noisy environments presents a limitation in MAV applications.

## Real time testing of handmade prototypes

### Design 1

**Figure 26:**
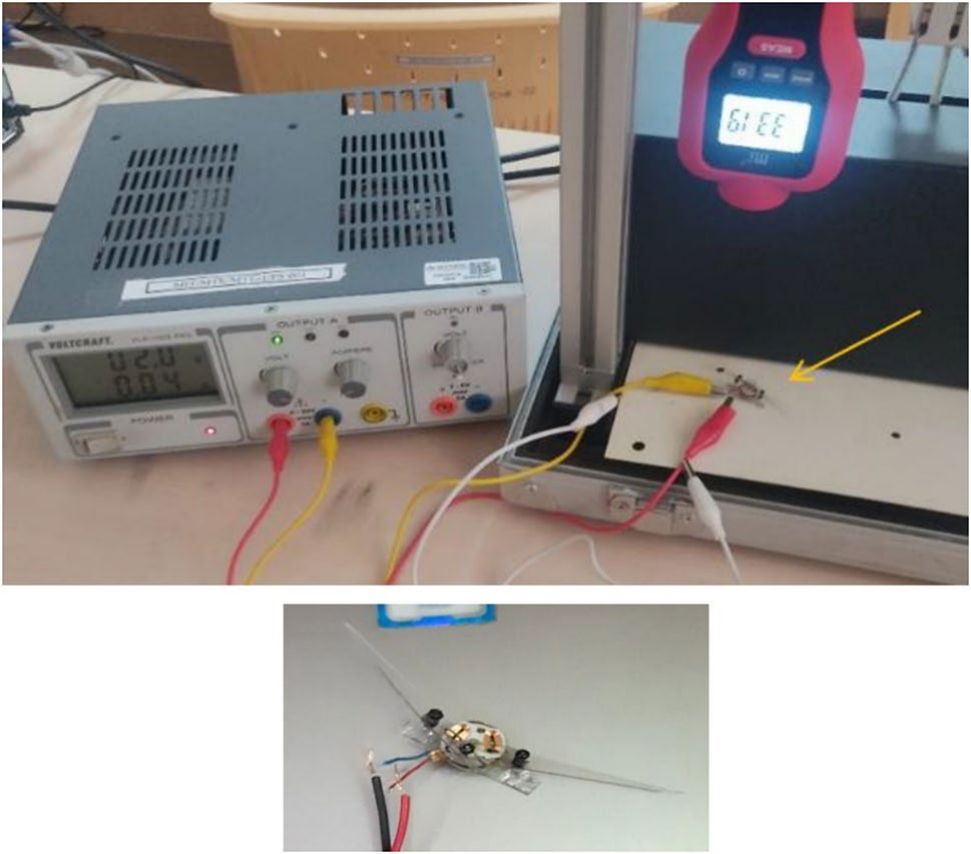
Homemade SC-SDL design-1 with a button motor frequency testing setup.

**Figure 27:**
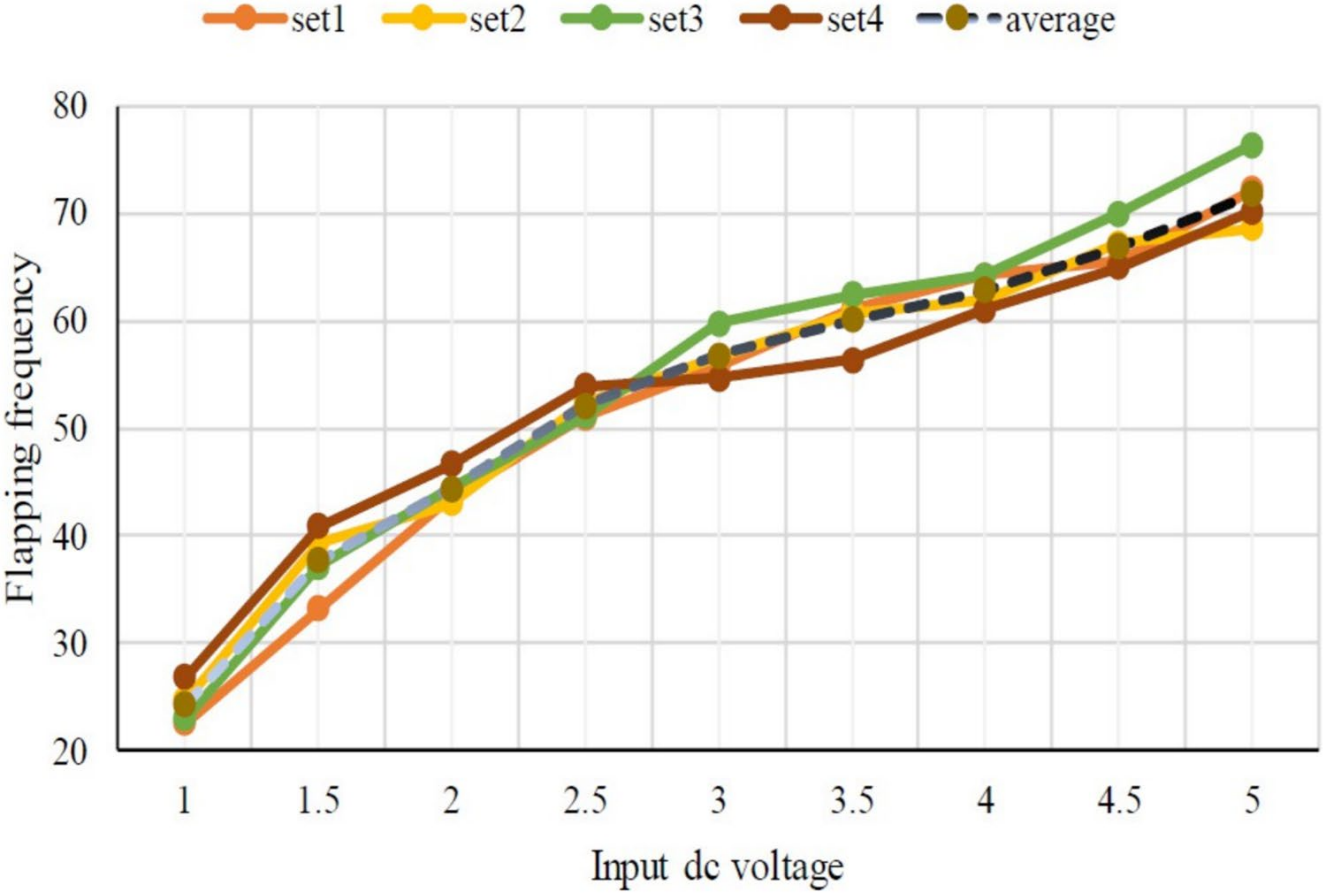
Obtained set of flapping frequencies from button motor driven SC-SDL Design-1 model.

**Figure 28:**
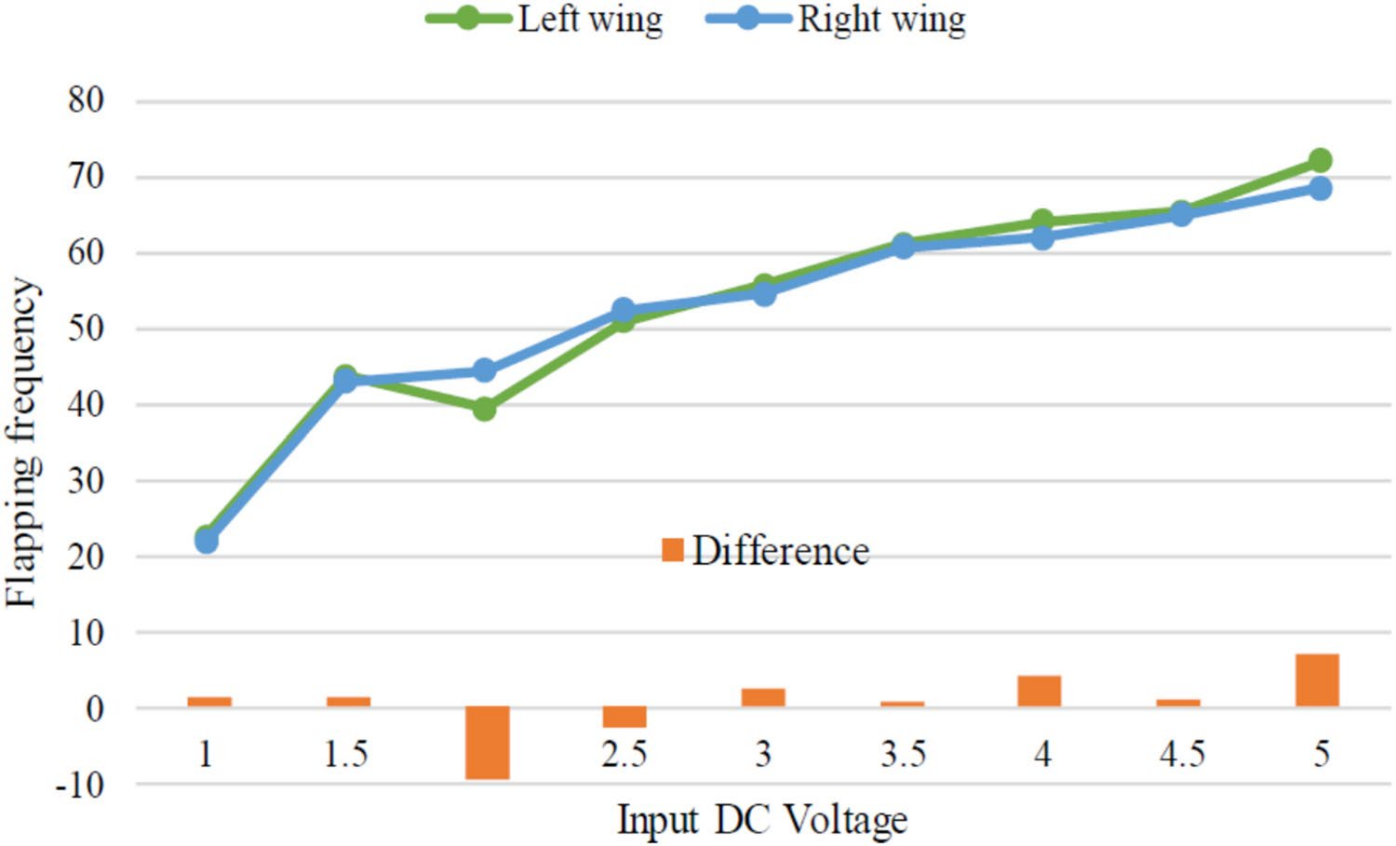
Difference of flapping frequency obtained between left and right wing of button motor driven SC-SDL design-1 model.

**Figure 29:**
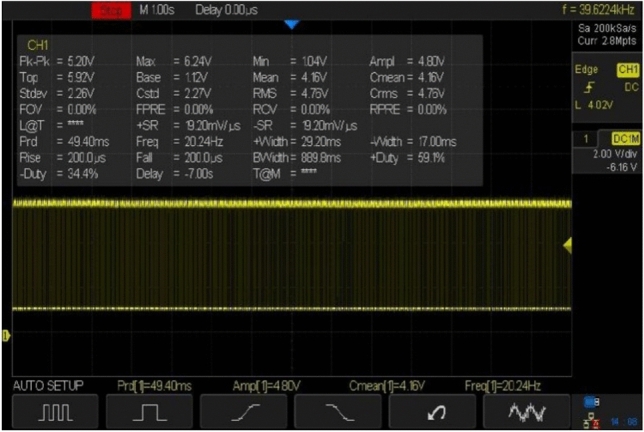
IR sensor measured frequency measurement of handmade SC-SDL design-1 mechanism with Button vibration motor design at 1v.

At first, we experimented with a hand-made version of the SC-SDL concept by attaching a single button vibration motor to both the links, as illustrated in Fig. 26. The total weight of the mechanism design with the button vibration motor but without wings has reached 0.88 g. As per our literature survey, 0.88 g is the least amount of mass of any of the currently available motor-actuated flapping wing mechanisms with motor and without wings design. The obtained weight of the micro DC motor-actuated design is 0.85 g. As Fig. 26 shows a homemade SC-SDL design with a button motor, the same design is connected to a variable DC power supply and a laser tachometer so that the flapping frequency can be measured and recorded separately depending on the supply voltage. When the input voltage is changed from 1 to 5 V, several sets of measurements are produced, as shown in Fig. 27; the highest flapping frequency obtained is 76.3 Hz when the voltage is 5 V. The left- and right-wing flapping frequencies are measured, and the difference obtained is projected in Fig. 28. The obtained difference in flapping frequency is minor; hence, it can be considered negligible. The same flapping frequency verification was validated for 1 V by measuring it through an IR sensor, and it produced 20 Hz, as shown in Fig. 29.

### Design 2

**Figure 30:**
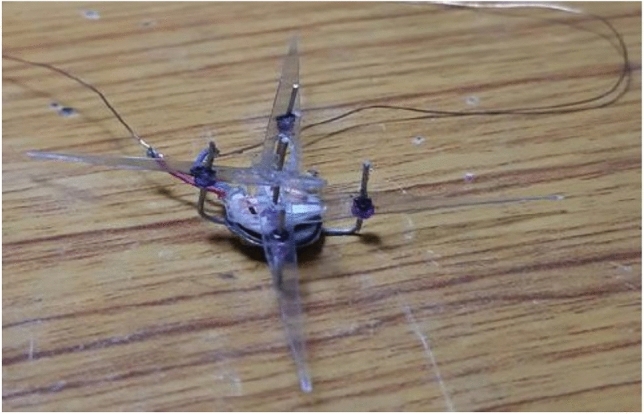
Handmade SC-SFL design-2 with button vibration motor (without wings).

By considering the same (Fig. 26) design-1, the modifications are implemented to obtain the (Fig. 30) design-2 which is similar to the simulations of Figs. 6 and 7. Design-2 is being constructed as a handmade design, as illustrated in Fig. 30, where 1.07 g it weighs.

**Figure 31:**
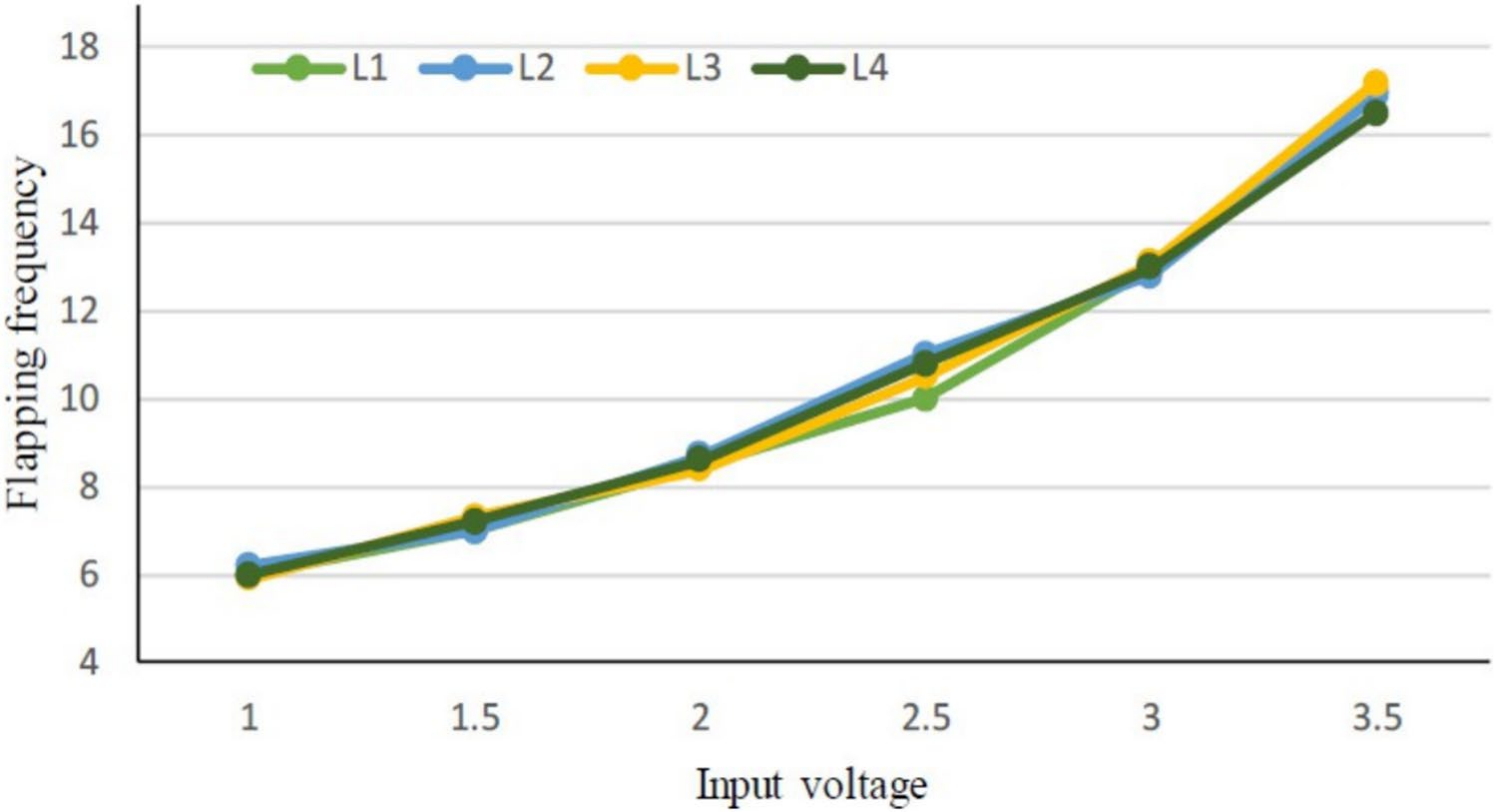
Obtained set of flapping frequencies from button motor driven SC-SFL Design-2 model.

**Figure 32:**
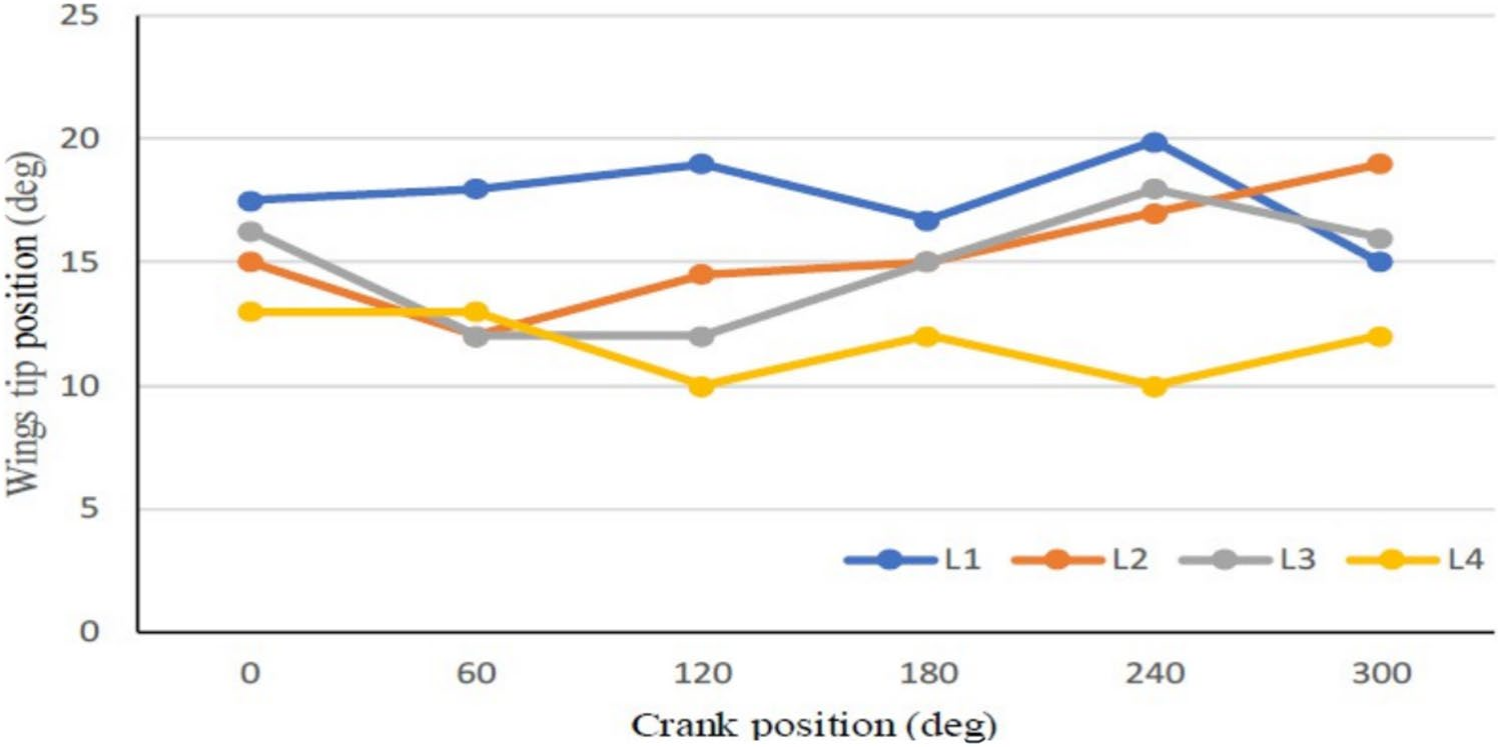
Wing tip positional variations of all the four handmade design -2 wings.

Increasing the number of sliding levers stacked on top of each other at the crankshaft of the button motor causes a decrease in its speed due to the added load. The obtained flapping frequency ranges for the SC-SFL design are between 5 and 18 Hz when the input DC voltage of 1–3.5 V is varied with respect to time. As it can be seen in Fig. 31, the flapping frequency of the SC-SFL design-2 is lower in comparison to the frequency of the SC-SDL design-1. Variation in all the four sliding levers can be observed in Fig. 32. A single motor crankshaft is driving four levers arranged in a 90-degree phase between each other. As the crank rotates from the starting position to different angles, the variations in the angular position of all four levers are depicted in Fig. 32 in detail. Due to the lack of coordination of wings in design-2, the modifications of design-1 produced the design-3 concept, where each individual lever is controlled by an individual motor for ease of control and coordination of both levers. Real-time experimental analysis is illustrated in the next section.

### Design 3

Design-3 concept was conceived as a result of the limitations in design-2 and 3, where wing coordination was lacking. In response, modifications from design-1 were integrated to create a new concept. Design-3 features individual lever control, each powered by its own motor. This setup enhances ease of control and coordination between the levers, addressing the shortcomings of previous designs. In this section, emphasis is placed on prioritizing individual wing control, leading to a detailed exploration of the selected hardware components tailored for this purpose. Effectively handling intermittent disruptions like air turbulence or wind is vital for ensuring stable flying in small and micro air vehicles, particularly for Flapping Wing Drones. Adaptive control systems can dynamically modify the control parameters in real-time to counterbalance variations in flying conditions. Furthermore, robust feedback control systems, such as Proportional-Integral-Derivative (PID) controllers, can be adjusted to effectively manage disturbances. A handful of researchers have employed Kalman filters to assess the status of a drone in the face of noise and disturbances. This can be achieved by combining predictions from the drone’s model with actual sensor measurements. The sophisticated technique involves using predictive algorithms to anticipate disturbances and proactively change the flight route. Upon meeting an unexpected gust of wind, the system would promptly identify the disruption by utilizing its IMU (Inertial Measurement Unit) and optical flow sensors. The Extended Kalman Filter (EKF) would yield a precise estimation of the drone’s current condition. This estimation would then be utilized by the adaptive Proportional-Integral-Derivative (PID) controller to regulate the flapping frequency and amplitude, therefore achieving stabilization of the drone. Concurrently, the Model Predictive Controller (MPC) would modify the trajectory to steer clear of the turbulent region, while the disturbance observer would optimize the control inputs to counterbalance the disturbance. The wings’ flexibility would passively mitigate the impact of the gust, guaranteeing seamless and steady flying. Flapping Wing Unmanned Aerial Systems can maintain steady flight and successfully handle infrequent disruptions by utilizing a combination of strong control algorithms, advanced sensor fusion, proactive path planning, and intelligent mechanical design. Hence an attempt of PID based controlling strategy has been performed in this paper.

*Hardware*: The components ESP8266 microcontroller, custom-made IR sensors, TB6612FNG motor driver, micro dc motor, and Duracell 9v alkaline battery have been integrated to make this prototype controlable.

*Software*: Using a joystick API created from scratch that transmits X and Y coordinates wirelessly via WiFi commu- nication protocol to ESP8266. This microcontroller of the bot with two motors (left motor and suitable motor) maps joystick coordinates (x values and y values) to Pulse Width Modulation (PWM) values for each motor. These PWM values determine the speed and direction of each motor, which ultimately controls the bot’s flight. A Proportional Integral Derivative (PID) algorithm^47^ is employed with the flight controller that registers the current rpm of each motor using an IR sensor and enhances its performance according to the Setpoint that the joystick API provides through the X and Y coordinate^48–51^.

*Joystick Coordinates*: The joystick coordinates are represented by x values and y values, where x values represent the horizontal position, and y values represent the vertical position of the joystick. These coordinates typically range from -255 to 255.

*Mapping to PWM Values*: The code contains conditional statements that map joystick coordinates to PWM values for the left motor (pwm values a) and the suitable motor (pwm values b). These conditional statements define how the bot should behave in different regions of the joystick.

*Center (Neutral)*: If both x and y coordinates are within the range of -5 to 5, the PWM values for both motors are set to This represents a neutral position where the bot should remain stationary.

*Forward and Backward*: If x is within -5 to 5 and y is outside this range, the code maps the y coordinate to PWM values for both motors. This controls forward and backward movement.

*Right and Left*: If y is within -5 to 5 and x is outside this range, the code maps the x coordinate to PWM values for both motors. This controls right and left movement.

*Top Right, Top Left, Bottom Right, Bottom Left*: These regions correspond to diagonal movements. The code contains more complex conditional statements to handle these cases, considering both x and y coordinates to determine the PWM values for each motor.

*PWM Values*: The PWM values are determined using the map to pwm function. This function takes the input joystick coordinates and maps them to PWM values within a specified range (0 to 1023). The PWM values control the speed and direction of the motors.

**Figure 33:**
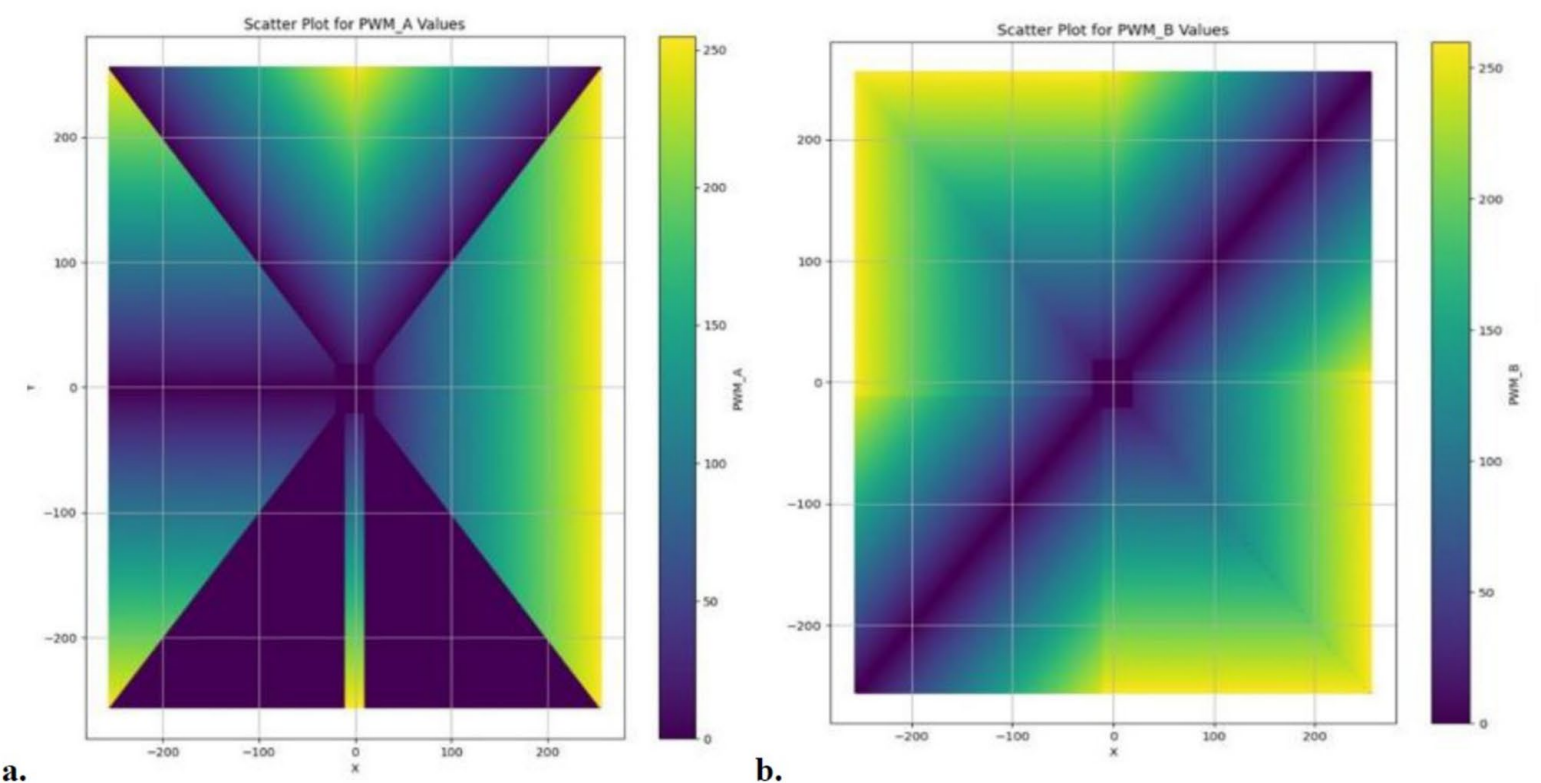
Scatter plots to visualize PWM values for A and B are assigned to different joystick coordinates.

*Scatter Plots*: The obtained code created scatter plots of both side motors (Fig. 33) provides detailed visualization of PWM values that are assigned to different joystick coordinates. Each plot shows the distribution of PWM values for one of the motors (pwm values a or pwm values b) based on the joystick coordinates.

**Figure 34:**
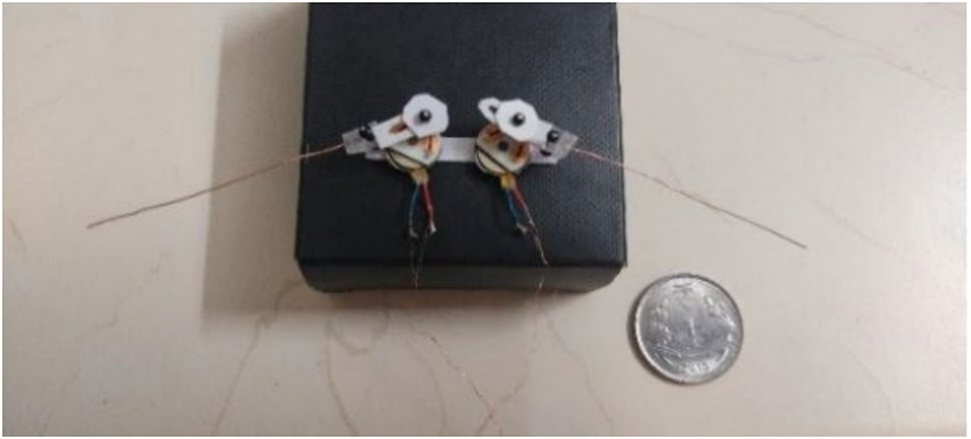
Handmade prototype model of Dual Crank – Slotted Dual Leavers (DC-SDL) model (DESIGN-3) with dual button vibration motor.

*Implementation details*: For the control of the handmade prototype model Fig. 34, Python is used to design the joystick API, and Micropython language is used to code into the esp8266 microcontroller. VScode with the Pymakr plugin is the framework used to upload code in esp8266, and Jupyter Notebook is used for the PWM visualization of the flight control system.

*Maneuvering and Control of Design -3 Through Motors*: When the joystick is in the center (neutral position), both motors receive PWM values of 0, causing the FWD to remain stationary. When the joystick is pushed forward or backward, the FWD will move forward or backward, respectively, with the speed determined by the joystick’s vertical position.

**Figure 35:**
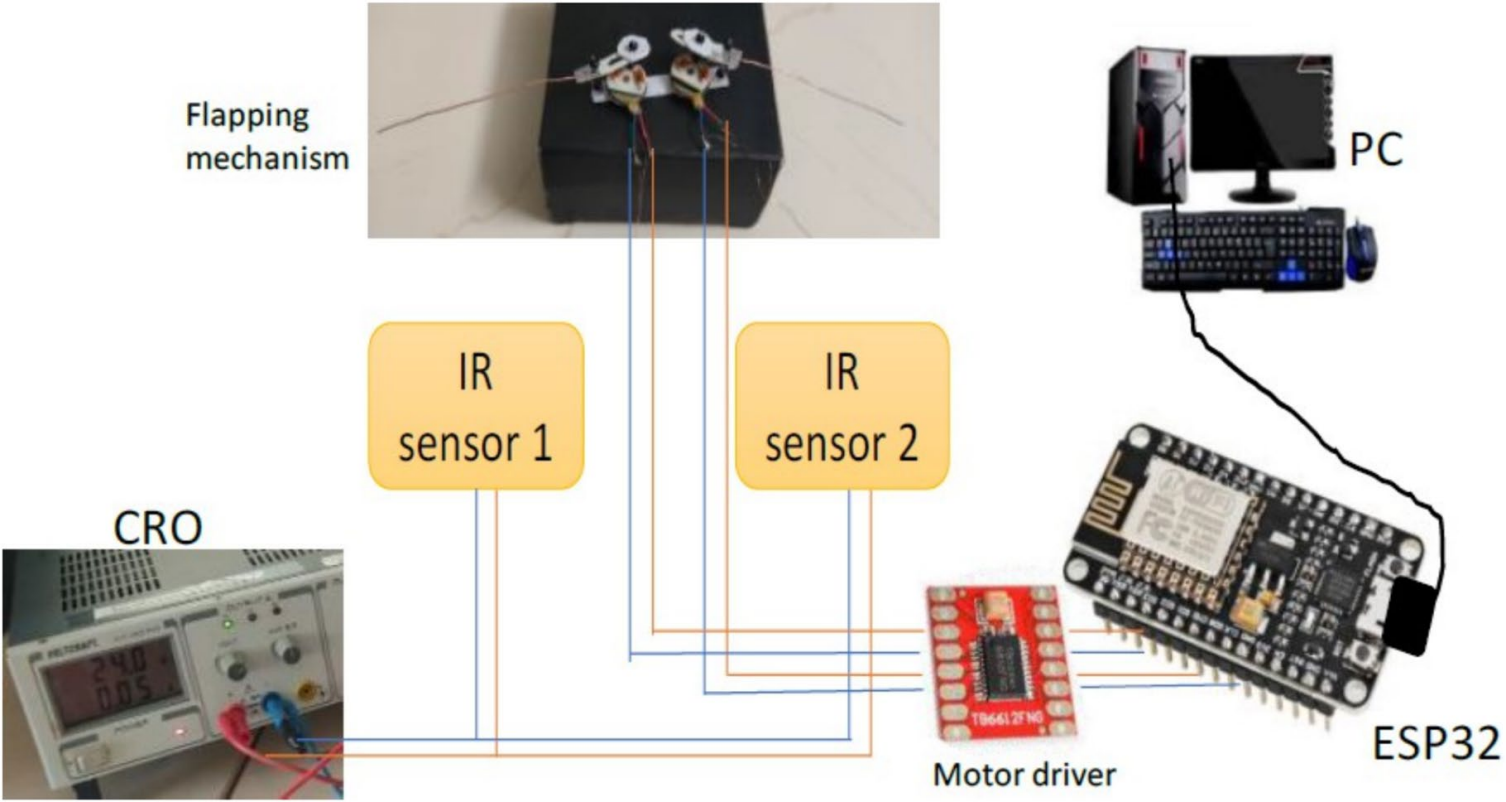
Experimental setup to validate the maneuvering of the prototype design-3.

When the joystick is pushed to the right or left, the bot will turn right or left, respectively, with the speed determined by the joystick’s horizontal position. Diagonal movements are also supported, allowing the FWD to move diagonally based on the combination of joystick coordinates. The conditional statements and PWM mapping logic in the code determine the specific behavior within each region. Overall, maneuvering and control is a crucial part of a flight controller system, enabling precise control of the FWD movement based on joystick input. The FWD flight behavior is determined by the mapping of joystick coordinates to PWM values, as defined in the code, and the scatter plots help visualize this mapping for analysis and fine-tuning. The experimental setup created for the validation of the left, right and forward movement of the design-3 prototype is as shown in Fig. 35.

**Figure 36:**
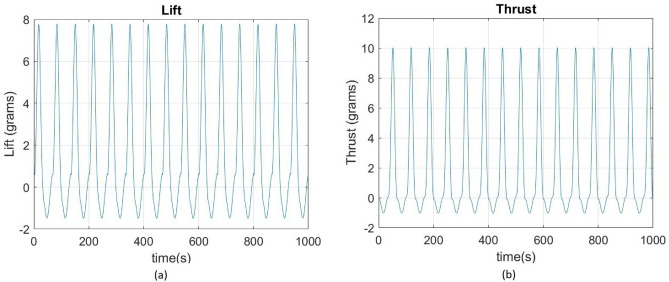
(**a**) Lift Variation with time, Lift Avg. = 5.265 g, (**b**) Thrust Variation with time, Thrust Avg. = 2.936 g.

By referring to modified strip theory and considering the specifications as in Table 5, the results obtained from the current simulations are as shown in Fig. 36. It indicates that when an average power of 1 W is provided to the proposed model, and the average flapping frequency is set at 130 Hz, it will provide an average lift of 5.265 g and an average thrust of 2.936 g. Given that the lift created by the suggested wing is imperative, the total weight of the full MAV (including the batteries, wings, and mechanics) must be lower than that value to accomplish the desired outcome. Therefore, the simulations indicate that to achieve a viable flying model, it is necessary to enhance the lift. This can be accomplished by either enlarging the wings or raising the flapping frequency. Hence, it is recommended to meticulously incorporate aerodynamic calculations into the wing design process. By implementing this approach, it is feasible to optimize the mechanism to provide increased lift, leading to superior overall performance. By incorporating aerodynamic principles into the design process, it is possible to create a flapping wing system that is more efficient and effective. This ensures that the lift generated by the system meets or surpasses the specified parameters.

**Table 5:** Assumed Specifications for the proposed design.

Parameter	Value
Flow velocity (*V*)	1.0 m/s
Span	80 mm, thin cambered line airfoil
Chord	14 mm at root
Reynolds Number	Based on local chord
Average frequency with wing	130 Hz
Max. flapping angle	49°
Kinematic viscosity (*ν*)	0.723e−5 m^2^/s
Angle of section’s zero-lift line (*α*_0_)	− 1.0°
Drag coefficient due to skin friction (*C*_*d f*_ )	0.080
Aspect ratio	0.683
Max stall angle (*α*_*stall*_)	18.0°
Efficiency (*η*_*s*_)	0.98

In the preliminary stage, the ESP32 microcontroller was configured to function as the central processing unit (CPU) for our motor control system. Wireless connections were established to facilitate the transmission of signals for both the X and Y axes of the joystick, while also assuring appropriate ground and voltage connections. Priority was also given to the integration of two infrared sensors, which functioned as tachometers to gauge motor speed. Precise connections were made between the output of these sensors and specific GPIO ports on the ESP32. Upon the effective connection of the motor driver module to the ESP32 microcontroller, the motor control system advanced. Connectors for PWM control signals, motor direction control, and critical power connections were meticulously established. To guarantee accurate polarity, the motors were linked to the output terminals of the motor driver, which corresponded to the specified motor channels. We established an essential feedback loop by linking the infrared sensor to the output of the motor driver. The implementation of this closed-loop configuration enables the system to dynamically modify motor parameters in response to the motor speed in real-time, thereby augmenting overall control and efficiency. Compatibility between voltage source levels and the specifications of every component—including the ESP32 microcontroller, motor driver, and motors—was maintained through meticulous attention to detail. To streamline the processes of programming and monitoring, a USB/Serial link was established between the computer and the ESP32 microcontroller. This stage was critical in ensuring uninterrupted programming and instantaneous system monitoring. By harnessing the capabilities of the ESP32 and the MicroPython programming language, successfully transmitted a logical control program customized for motor operations, thereby guaranteeing the motor control system’s seamless operation for the circuit as in Fig. 35.

**Figure 37:**
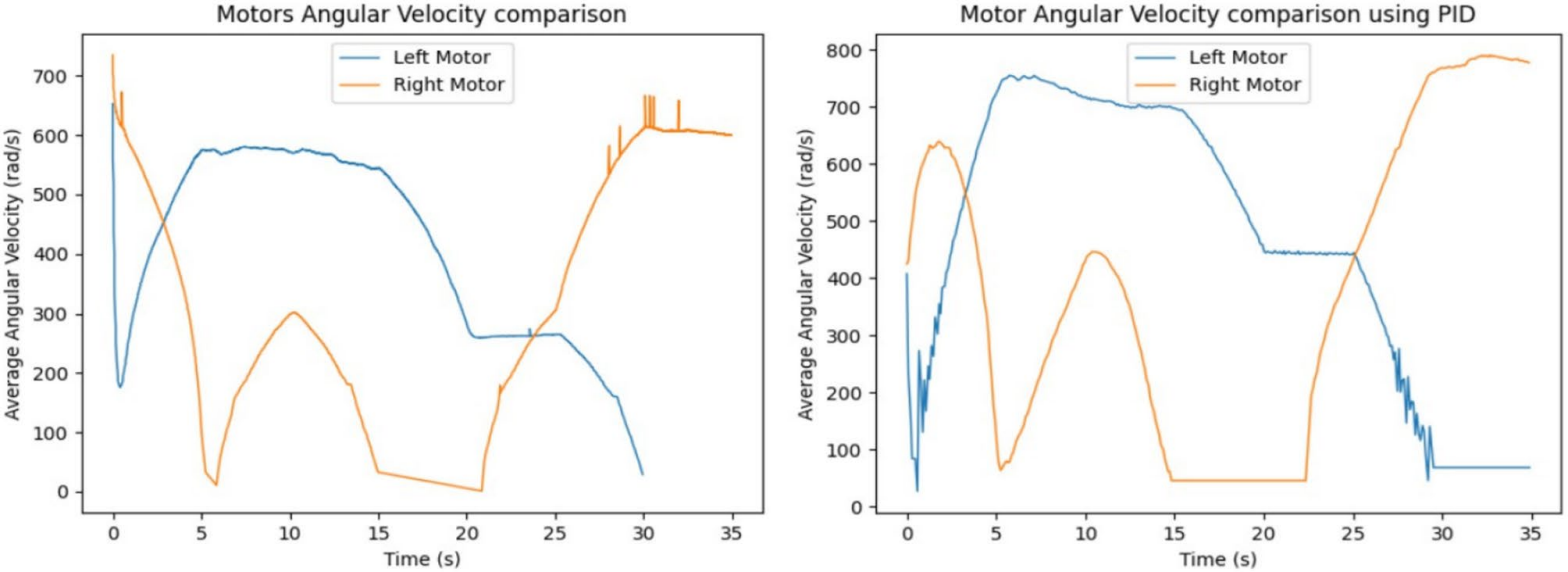
Real time angular velocity comparison of left and right motor connected at design 3.

**Figure 38:**
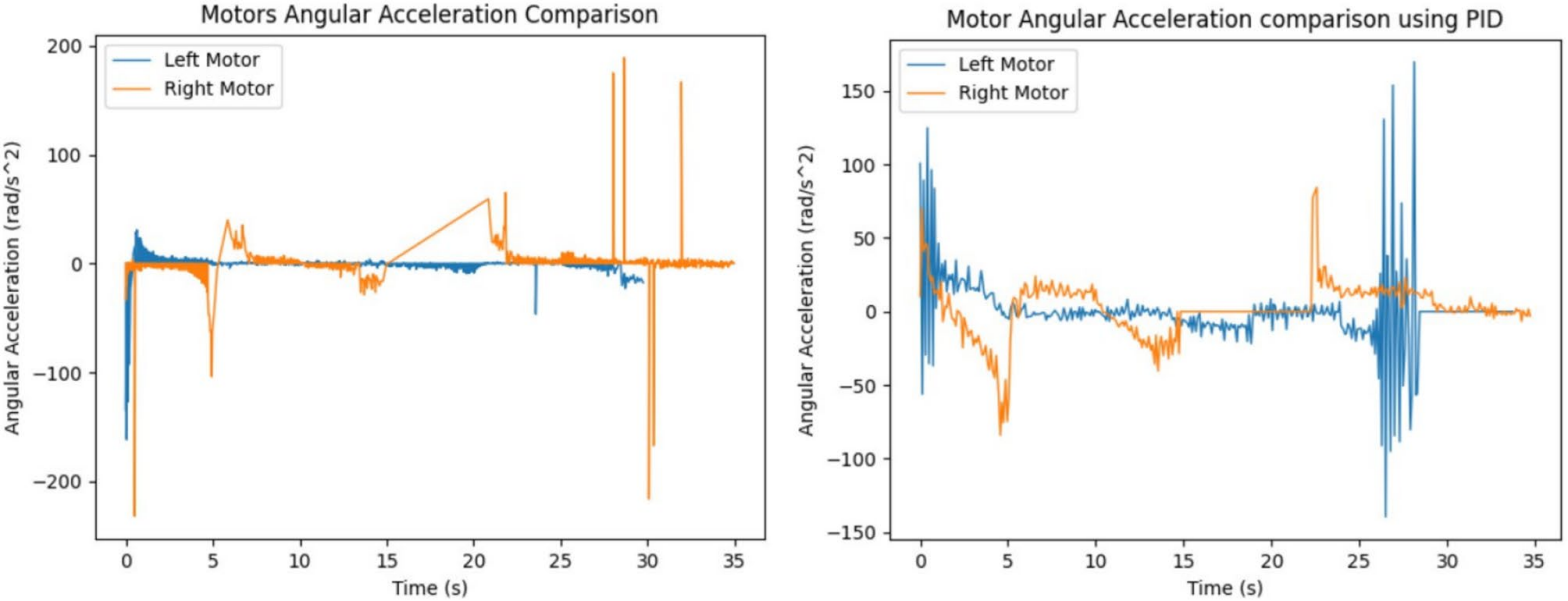
Real time angular acceleration comparison of left and right motor connected at design-3.

**Figure 39:**
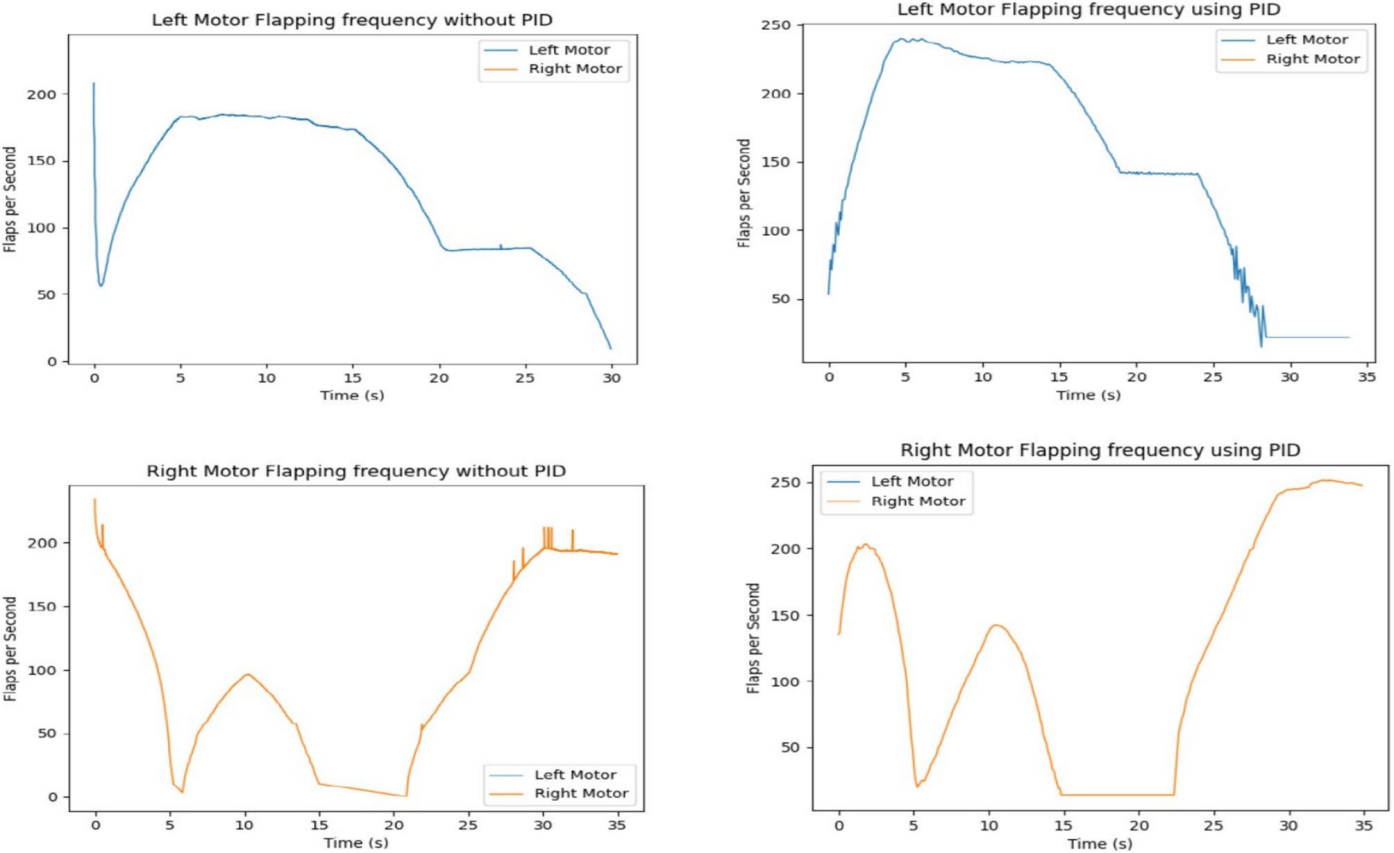
Real time flapping frequency comparison of left and right motor connected at design-3 (for without PID and with PID controller).

As shown in Figs. 12 and 13, the input velocity varies every 5 s as a supply source for Design 3. IR sensors are connected to both the left and right motors to analyze flapping frequency, angular velocity, and acceleration, validating the obtained simulation results. The input voltage follows the flowchart process illustrated in Fig. 13. During the first 5 s (i.e., between the 0–5 s time slot), a 3.7 V supply is applied to both motors. This ensures that the angular velocity remains the same for both the left (L) and right (R) motors, indicating forward movement at a constant speed. However, as shown in Fig. 37, the angular velocity variations are in opposite directions for the L and R motors since they rotate in opposite directions due to their supply connection polarity, as depicted in the right-inverted configuration of Fig. 15. The expected maneuvering of the proposed design, as demonstrated in Fig. 15, is validated through real-time results, confirming the controllability of the left and right wings. In real-time experiments, a motor requires some time to reach its maximum speed and maintain a constant velocity. However, since the direction changes every 5 s, an increase in speed at both motors is observed in Fig. 37. Additionally, Fig. 37 presents a comparison between the results without PID control and those with PID control, where the left motor is tuned with *K*_*p*_ = 0*.*09, *K*_*i*_ = 0*.*00, *K*_*d*_ = 0*.*002, and the right motor with *K*_*p*_ = 0*.*01, *K*_*i*_ = 0*.*003, *K*_*d*_ = 0. For the next 5 s (i.e., between the 5–10 s time slot), rightward movement is initiated by supplying 2 V to the R-motor while maintaining 3.7 V at the L-motor. This forward turn in the rightward direction results in speed, acceleration, and flapping variations, as shown in Figs. 34, 38, and 39. Specifically, Fig. 39 shows that the flapping frequency is 145 Hz at the R-motor and 230 Hz at the L-motor for a rightward turn. Furthermore, as depicted in Fig. 37, the L-motor experiences minimal variations in angular velocity between the 5 and 15 s time slot due to its constant velocity. In the subsequent 5 s (i.e., between the 10 and 15 s time slot), forward movement is resumed with both motors receiving a 3.7 V supply. However, due to prior supply variations at the R-motor, its angular velocity exhibits fluctuations in the opposite direction.

In the next 5 s (i.e., between the 15 and 20 s time slot), a leftward movement is initiated. The R-motor remains constant at 3.7 V, maintaining a steady speed, while the L-motor voltage is reduced to 2 V, resulting in a lower speed. This indicates a forward-leftward movement. For the next 5 s (i.e., between the 20 and 25 s time slot), a slow forward movement is executed by supplying 2 V to both motors, leading to a reduced forward speed. As shown in Fig. 33, both motors eventually reach a constant speed, intersecting at one point. However, after another 5 s, changes in voltage bring their speed to zero. Due to the opposite polarity of the motor connections, their angular speeds are in opposite directions, which is expected. According to Yonatan et al.^52^, during turn initiation in flapping-wing (FW) unmanned aerial systems, wing compliance generally reduces steering asymmetry, contributes to flight stability by dampening steering performance, and mitigates unwanted asymmetries caused by flight disturbances. Due to the structural asymmetry in wing coordination, the net lift is affected. Furthermore, during the downstroke phase, the airflow expands significantly, requiring maximum power to counteract the vertical lift force, thereby reducing propulsive efficiency. This imbalance in flow patterns indicates a shift in flapping power from propulsion to load suspension. The experimental investigations conducted on the proposed flapping-wing mechanisms provided valuable insights into the performance, limitations, and practical applicability of each design. The SC-SDL (Design-1) model, powered by a single button vibrator motor, successfully achieved symmetric flapping with a maximum frequency of 76.3 Hz at 5 V, demonstrating the viability of button motors for compact and lightweight actuation at insect scale. This design confirmed minimal frequency deviation between the left and right wings, validating the mechanical integrity of the crank-slider configuration. In contrast, the SC-SFL (Design-2) model, incorporating four stacked sliding levers driven by a single motor, exhibited a marked reduction in flapping frequency (5–18 Hz range) due to the increased mechanical load and coordination challenges. The lack of precise synchronization among the levers limited the lift generation and underscored the trade-off between design complexity and efficiency. To overcome these limitations, the DC-SDL (Design-3) design introduced dual independent button motors, enabling separate control of each wing. Experimental results demonstrated successful leftward, rightward, and forward maneuvers through controlled modulation of input voltages, with flapping frequencies reaching up to 230 Hz on one wing during differential actuation. The inclusion of PID control significantly enhanced system stability, reducing oscillations and improving response time during maneuvering tests. These findings highlight the potential of modular motor configurations and adaptive control strategies for achieving precise, stable, and efficient actuation in micro aerial vehicles. The experiments not only validated the simulation results but also provided a foundation for future refinement of flapping-wing mechanisms, emphasizing the need for further aerodynamic optimization and actuator selection to achieve sustained, untethered flight.

In summary, utilizing a button vibrator—by removing its weights to minimize vibrations and repurposing it as a motor to actuate wing flapping in forward (FWD) flight applications—is an innovative approach with both advantages and challenges. While this method offers benefits in terms of size, weight, and power efficiency, adapting button vibrators for continuous and controlled wing flapping requires addressing mechanical, control, and durability challenges. Extensive prototyping, testing, and potential integration with additional components may be necessary to achieve optimal flight performance for micro air vehicles (MAVs) or similar applications. However, certain considerations must be taken into account. Button vibrators are typically not designed for continuous operation as motors, and the mechanical wear and tear associated with prolonged flapping motion may affect their lifespan and reliability. While they consume low power, they may lack the torque or power output required for demanding flight scenarios, which could limit the vehicle’s ability to carry payloads or operate in challenging conditions. Additionally, their unique characteristics may necessitate custom mechanical adaptations to effectively translate rotational motion into the desired wing-flapping motion. Despite these challenges, button vibrators prove to be a highly suitable option for nano aerial vehicle (NAV) applications. As demonstrated in Fig. 39, their performance shows promising results even without considering wings mounted on sliding levers. Furthermore, the implementation of a PID controller has significantly improved their efficiency, making them a viable choice for lightweight aerial designs.

## Conclusion

This study presents a comparative analysis of different lever alignment designs implemented in insect-scale prototypes of Flapping-Wing Micro Aerial Vehicles (FWMAVs). These micro aircraft utilize fluttering wings powered by either single or dual miniature coreless DC motors, which operate on a minimal DC power supply. Various lever configurations are employed to establish an indirect connection with a simple crank-slider mechanism. The paper details the modeling and control of this slider-crank mechanism using SIMSCAPE Multibody in MATLAB. Additionally, simulation validations are performed using Compmech GIM software to analyze structural movement in both 3D and 2D, alongside CAD design modeling. The study examines variations in lift and thrust based on predefined criteria, analyzing their effects on different work surfaces. The results demonstrate the effectiveness of Proportional-Integral-Derivative (PID) and Self-Regulatory Fractional Fuzzy Control (SRFFC) strategies in regulating the speed and stability of interconnected DC motors under external disturbances. Notably, the SRFFC controller outperforms the Fractional PID (FPID) controller by providing superior disturbance rejection, smoother settling behavior, and overall system stability. These findings are validated through both simulations and real-time assessments, including maneuvering tests for leftward, rightward, and forward movements of the flapping-wing Unmanned Aerial System (UAS). By integrating simulation results with real-time evaluations of control strategies, this research makes a significant contribution to the advancement of maneuverable and efficient micro air vehicles. The study enhances the understanding and optimization of flapping-wing UAS configurations, emphasizing the importance of size, weight, and power efficiency while maintaining precise control and stability. The comparative analysis of control strategies highlights the robustness of SRFFC in applications requiring high stability and effective disturbance management. Ultimately, this research lays a strong foundation for future advancements inflapping-wing technology, expanding the capabilities of FWMAVs in next-generation aerial applications. While this study demonstrates the feasibility of driving a slider-crank flapping mechanism with an off-the-shelf button vibrator, a key limitation is that the commercial motor caps both the maximum flapping frequency and available torque, making it less suitable than dedicated brushless or piezoelectric actuators for high-performance flight.

## Data Availability

The datasets used and/or analysed during the current study are available from the corresponding author on reasonable request.
